# Supplementary material for: Human RAP1 specifically protects telomeres of senescent cells from DNA damage
Source: EMBO Rep. 2020 Feb 25;21(4):e49076. doi: 10.15252/embr.201949076 (PMC7132343; doi:10.15252/embr.201949076)
Supplement: Supplementary file 1 — Expanded View Figures PDF [file EMBR-21-e49076-s001.pdf]

Expanded View Figures

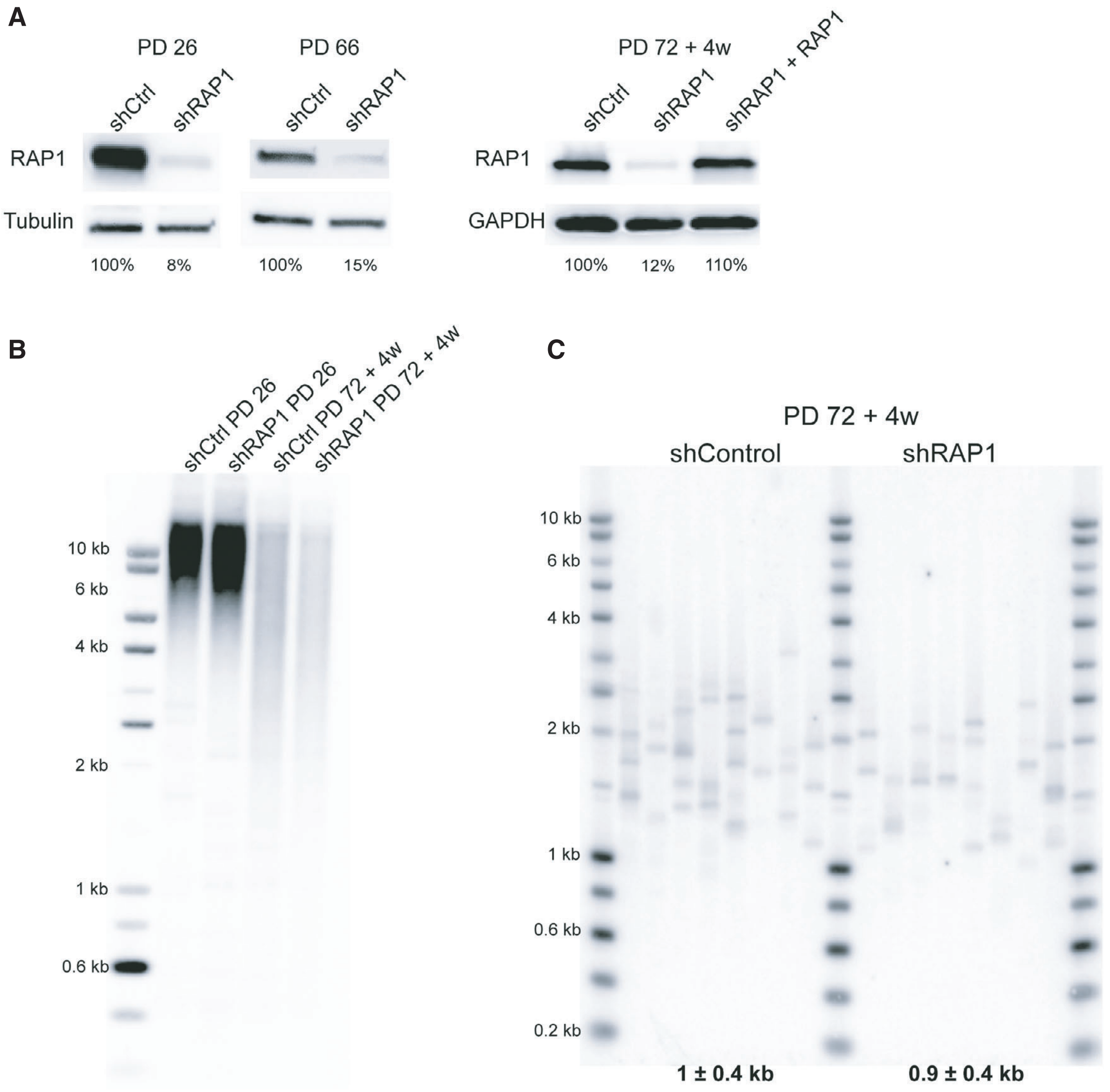

Figure EV1. RAP1 depletion does not affect telomere length.

A RAP1 expression of dividing MRC-5 cells (left panel; PD 26 and PD 66) and senescent cells (right panel; PD72 + 4 weeks). RAP1 depletion (or ectopic expression—right panel) was carried out for 10 days.

B Telomere restriction fragment of young (PD 26) and senescent cells (PD 72 + 4 weeks) upon 10-day shRAP1 incubation.

C Single telomere length analysis (STELA) of MRC-5 senescent cells transduced with either a control vector or an shRAP1-expressing vector. STELA was performed at the Xp/Yp telomere, and the mean telomere length ± SD, after subtracting 406 bp of flanking DNA, is shown at the bottom of the blot.

© 2020 The Authors

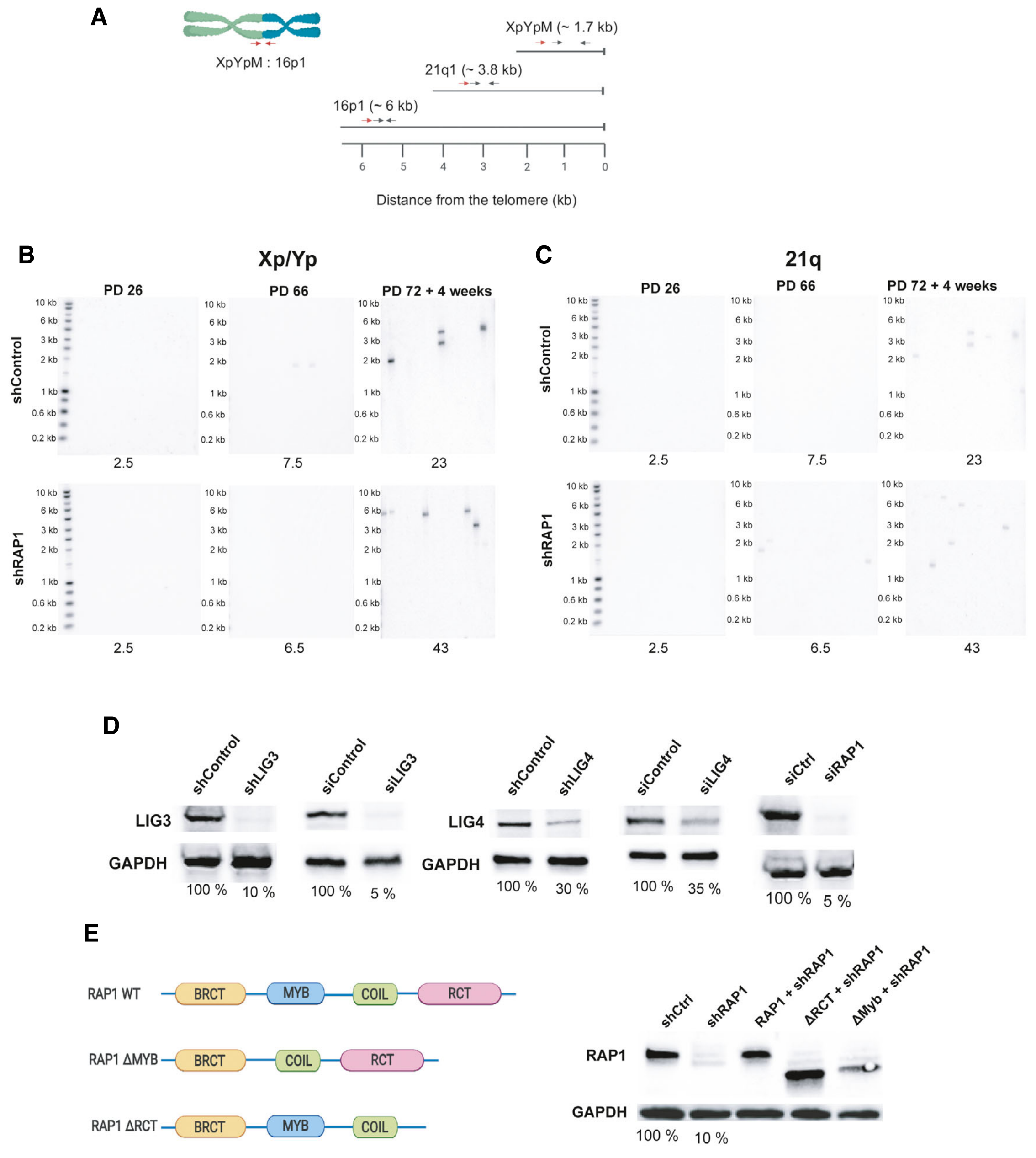

Figure EV2. Telomere fusion assay by PCR.

A Location of the primers and probes used for the telomere fusion PCR: Red arrows indicate positions of subtelomeric PCR primers, while black arrows represent the primers used to generate DNA probes for radioactive hybridization with the Southern blot membranes.

B, C Representative Southern blot membranes of the telomere fusion PCR assay shown in Fig 2A and B. Membranes were subsequently hybridized with the 16p probe, XpYp probe, and finally with the 21q probe.

D Different RAP1 constructs used in the telomere fusion assay of senescent MRC-5 cells. The percentage of protein expression is indicated below the blots.

E Expression of RAP1, LIG3, and LIG4 related to Fig 2C. The percentage of protein expression is indicated.

 © 2020 The Authors

Figure EV3. Telomere length and overhang are not affected by RAP1 depletion in HeLa cells.

A Telomere signal profile of cells treated with or without BIBR1532 +DOX as shown in Fig 4B. The size in kilobases of the highest peak is indicated.
B STELA assay at the Xp/Yp telomere of HeLa cells treated with the telomerase inhibitor BIBR1532 and depleted for RAP1 (+DOX). The scatter plots show the distribution of STELA products after subtracting 406 bp of flanking DNA, while error bars represent the mean ± standard deviation.
C Relative mRNA levels of *LIG3* and *LIG4* measured by RT–qPCR corresponding to the experiment shown in Fig 4C. Data represent mean ± SD of three independent experiments (***P = 0.0001 for shLIG3 and **P = 0.0061 for shLIG4; two-tailed Student's t-test).
D Protein expression of LIG3 and LIG4 used in Fig 4C. The percentage of protein expression is indicated.
E Telomere overhang assay in HeLa cells. In-gel hybridization of the telomere probe in native and denaturing conditions. On the right, quantification of the normalized telomere overhang signal performed by dividing the signal intensity obtained by native gel hybridization to the total signal obtained with denatured gels. Data represent one biological replicate.

© 2020 The Authors

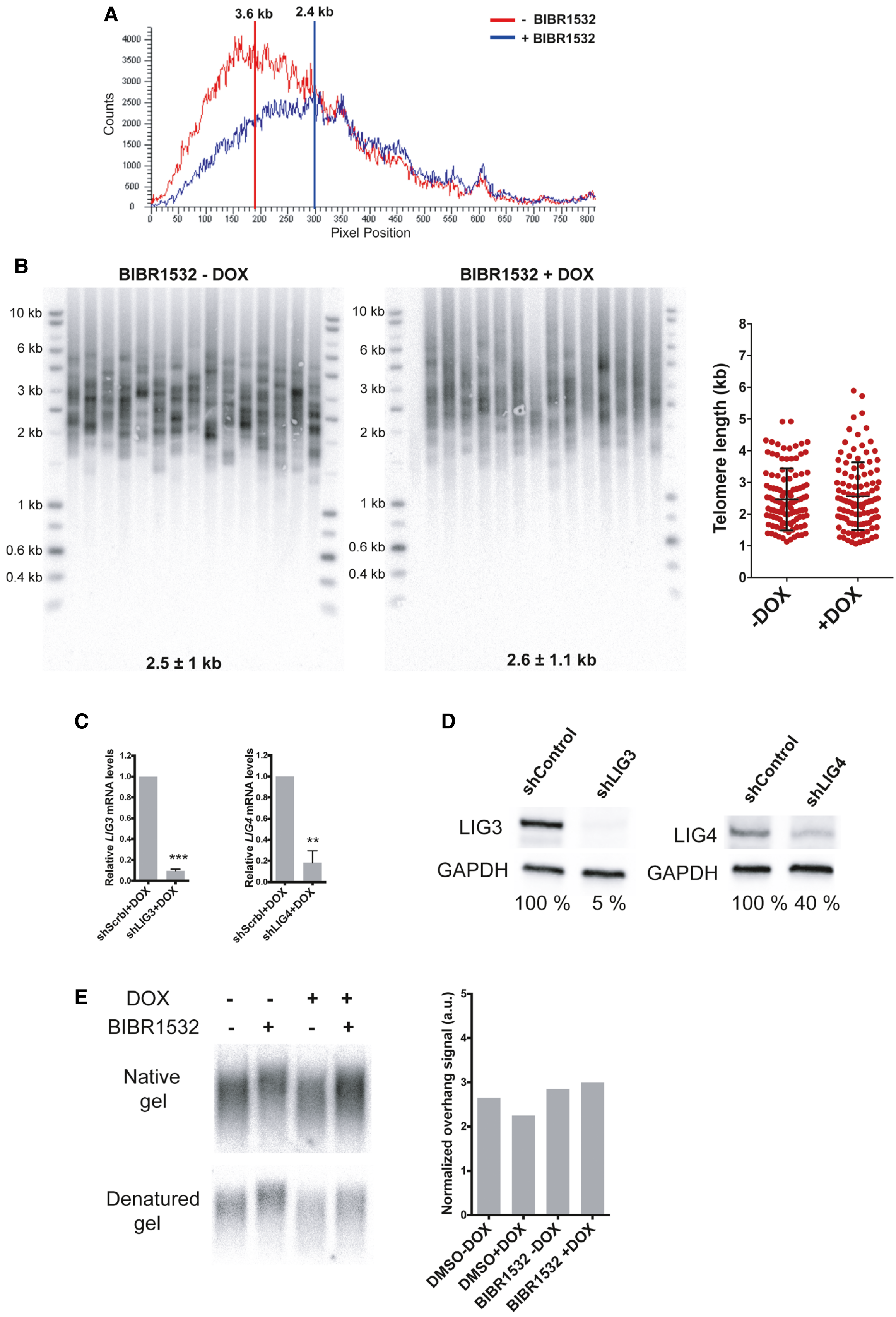

Figure EV3.

© 2020 The Authors
