# Supplementary material for: Human RAP1 specifically protects telomeres of senescent cells from DNA damage
Source: EMBO Rep. 2020 Feb 25;21(4):e49076. doi: 10.15252/embr.201949076 (PMC7132343; doi:10.15252/embr.201949076)
Supplement: Supplementary file 4 — Source Data for Figure 1 [file EMBR-21-e49076-s003.pdf]

Figure 1

a

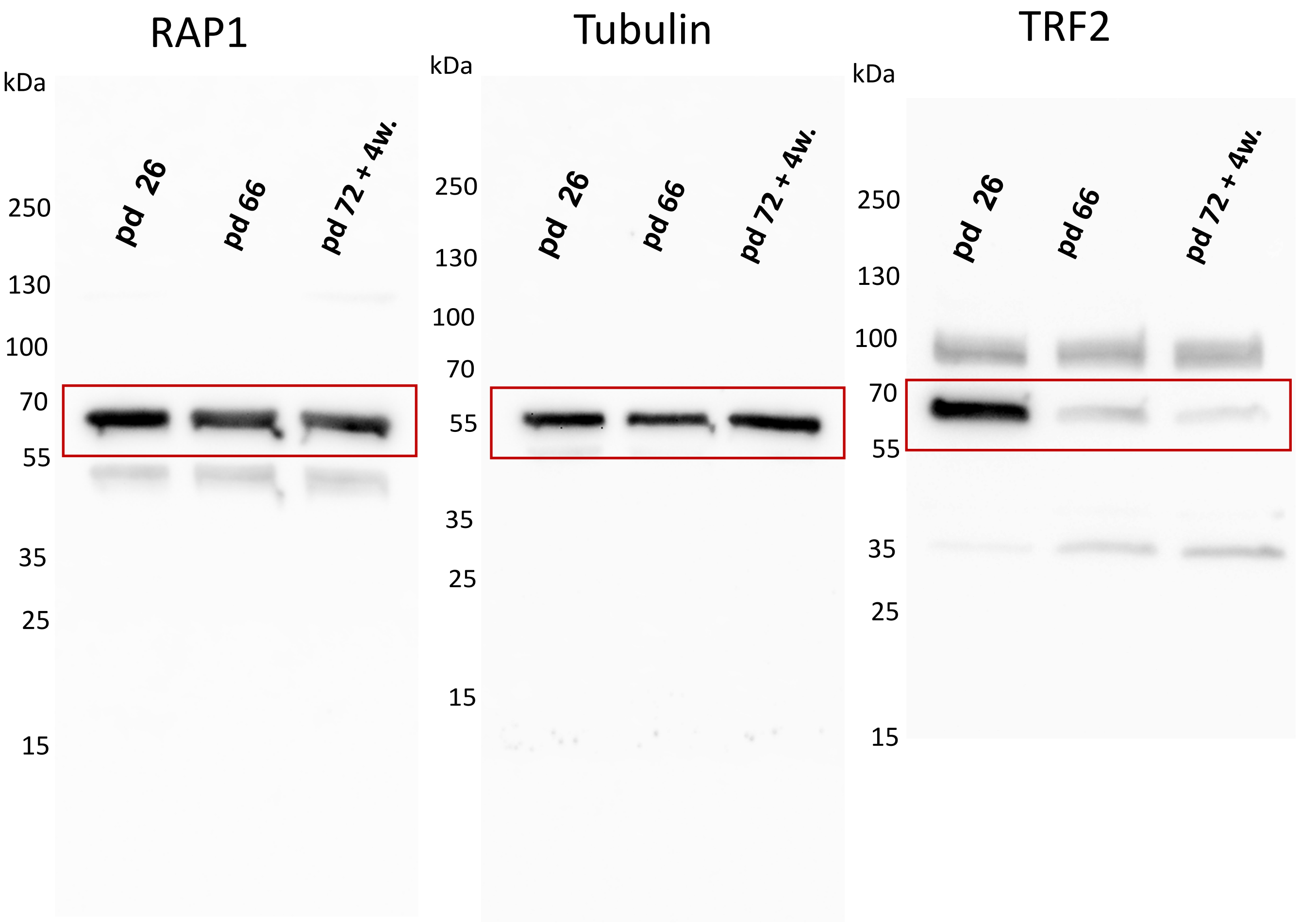
RAP1
Tubulin
TRF2
kDa
250
130
100
70
55
35
25
15
pd 26
pd 66
pd 72 + 4w.

Figure 1

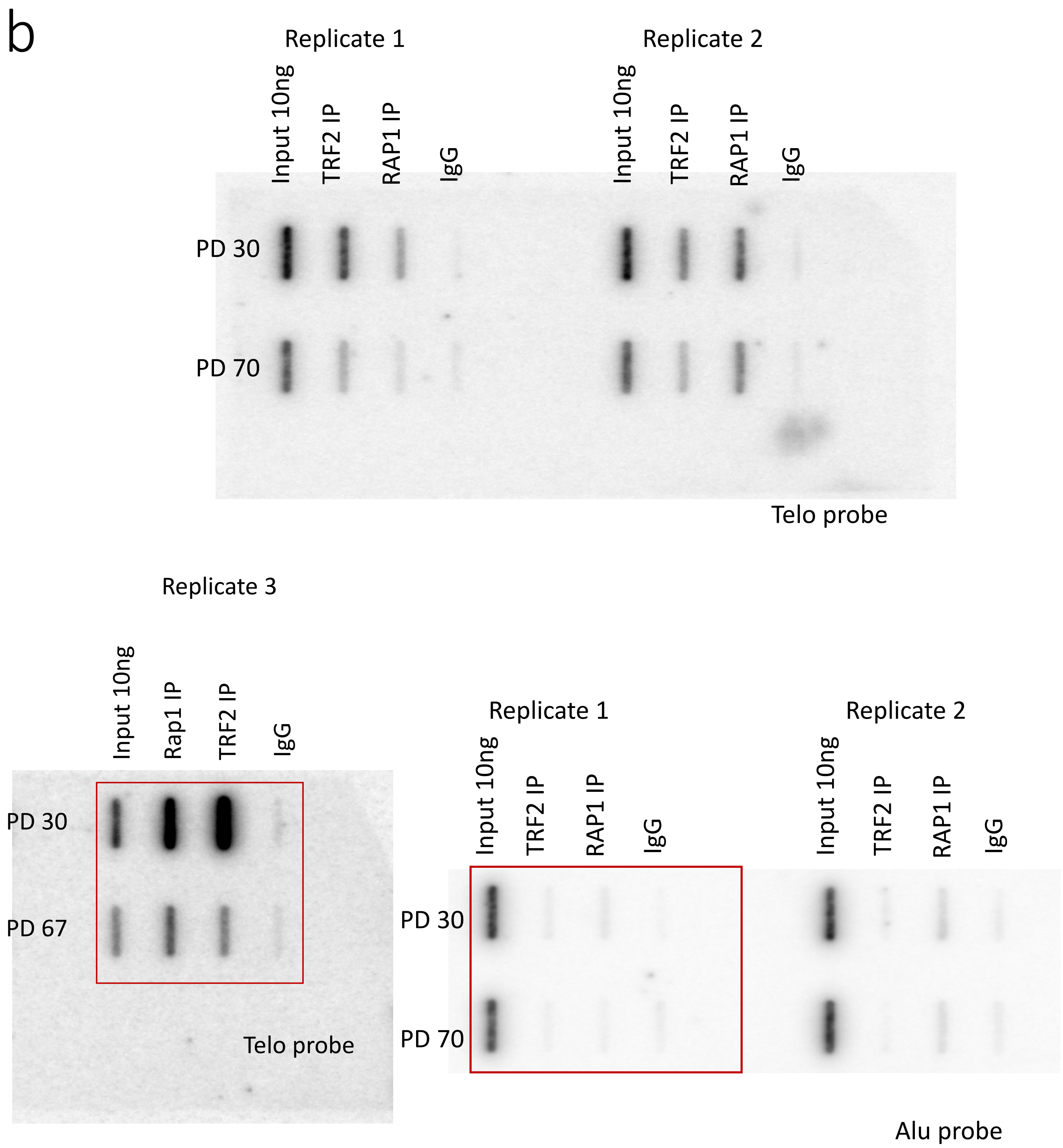
b
Replicate 1
Replicate 2
Input 10ng
TRF2 IP
RAP1 IP
IgG
PD 30
PD 70
Telo probe
Replicate 3
Rap1 IP
PD 67
Alu probe

Figure 2

a-b Replicate 1 (as in the main Figure)

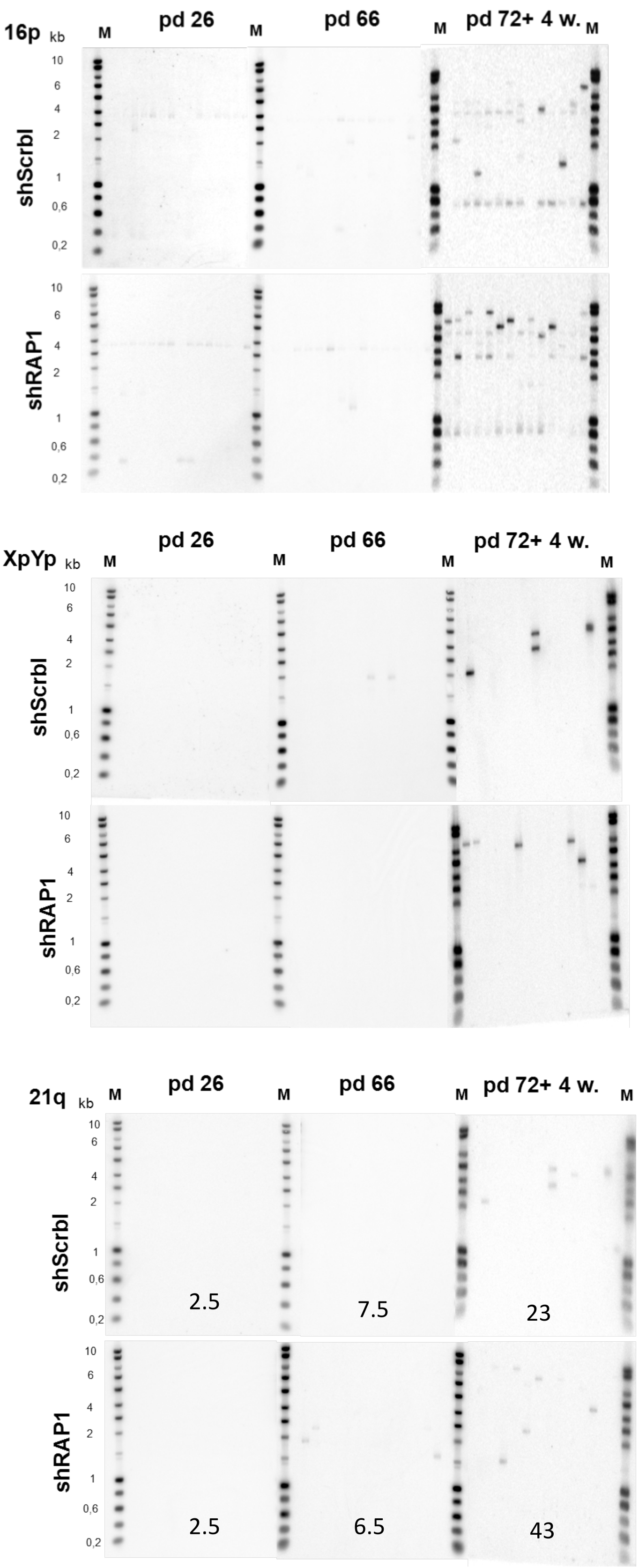

Figure 2
a-b Replicate 2

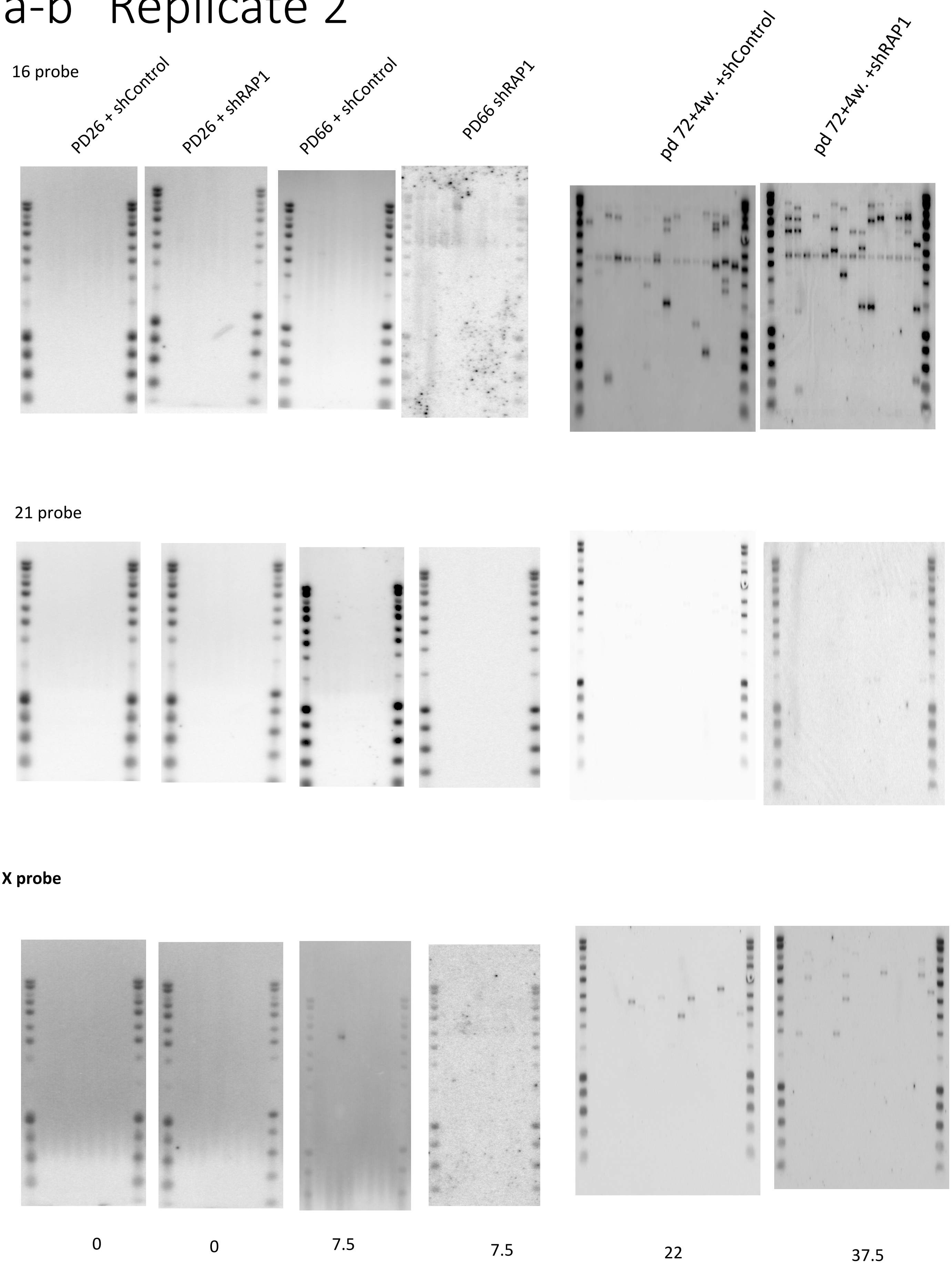

Figure 2
a-b Replicate 3

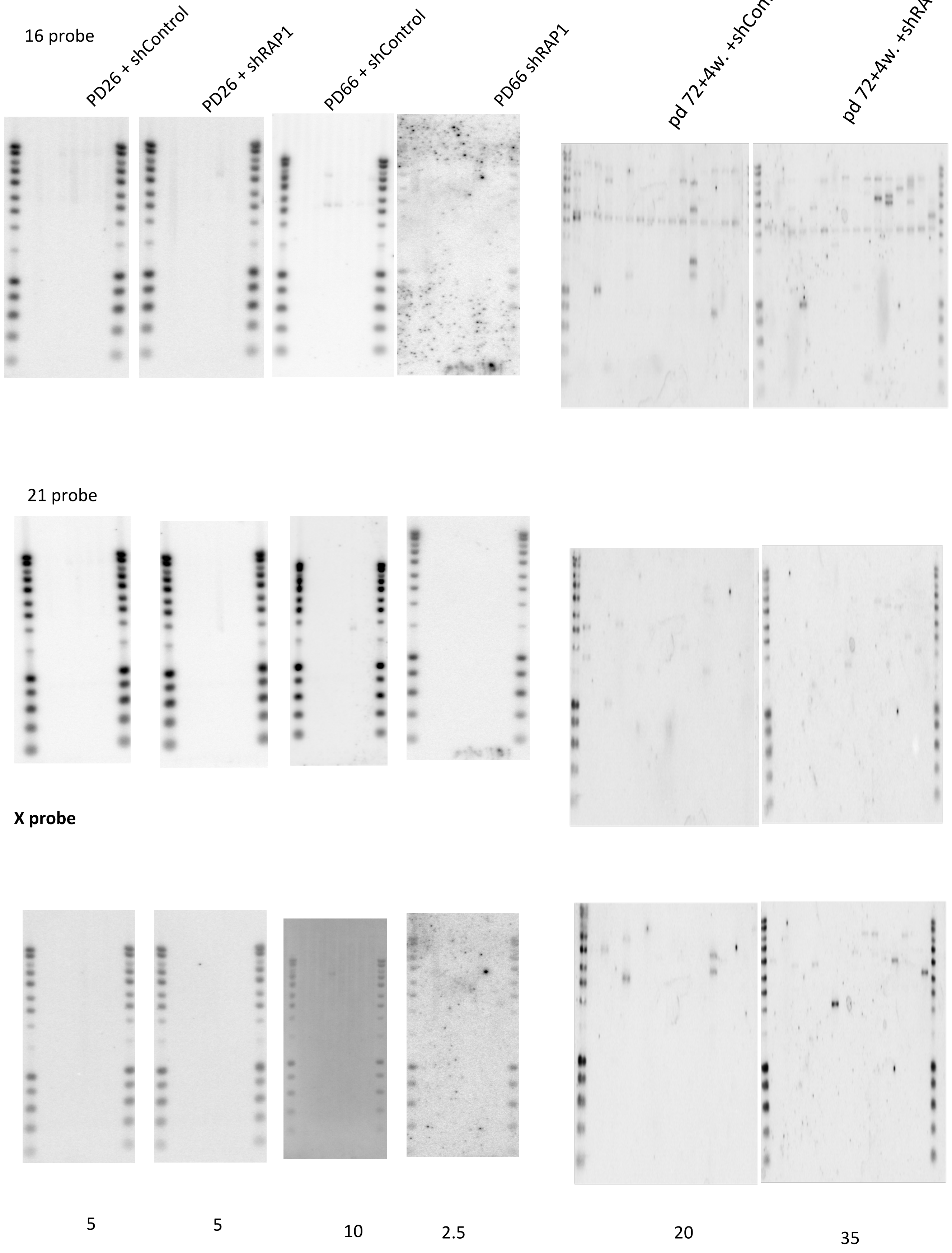

Figure 2
c-d   Replicate 1

16p **shRAP1+shLIG3** **shRAP1+shLIG4**

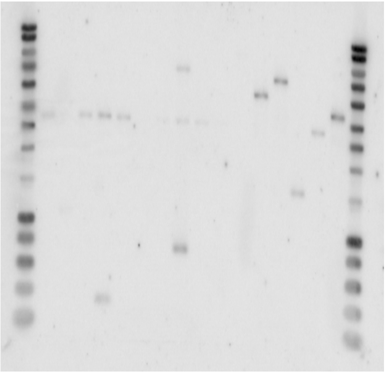

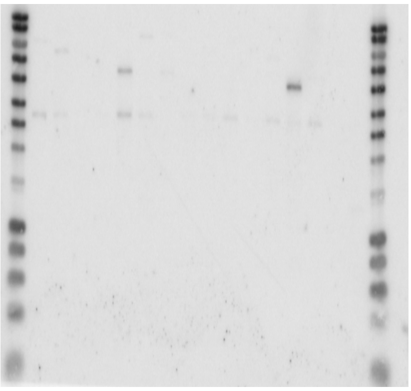

21q

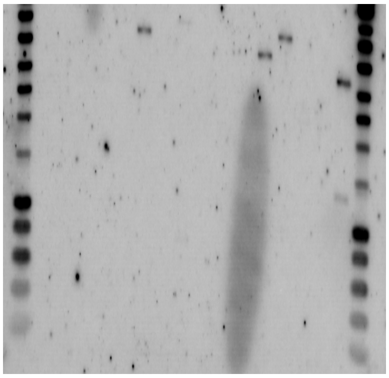

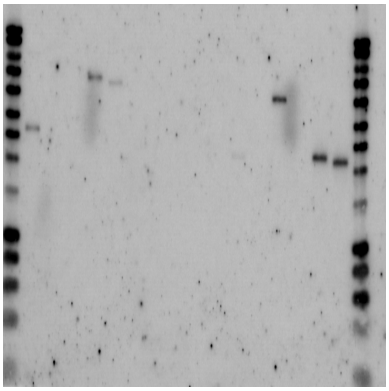

XpYp

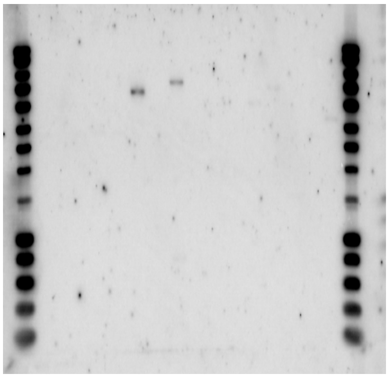

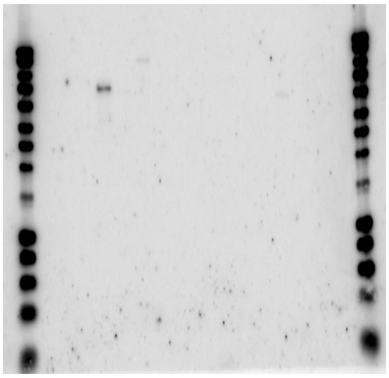

35

22.5

25 ng of DNA per 1 PCR

Figure 2

Replicate 2

16 probe

shLIG3+shRAP1 shLIG4+shRAP1

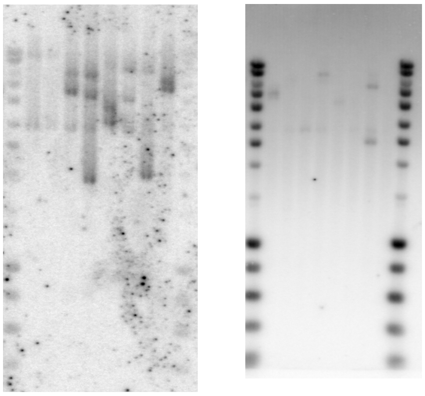

21 probe

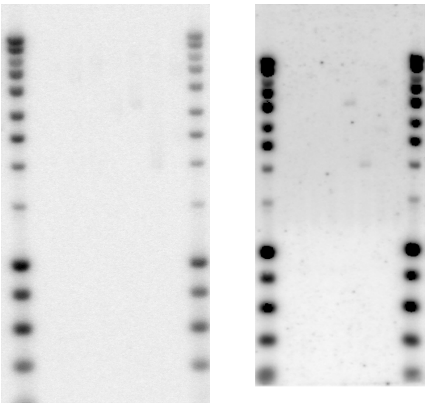

X probe

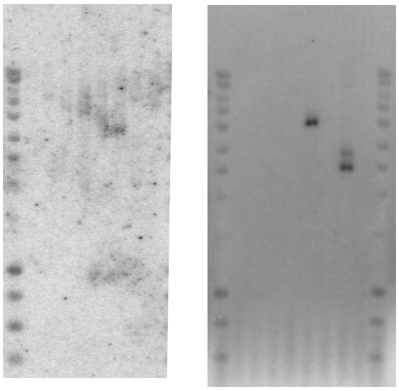

27.5 20

Figure 2

Replicate 3

16 probe

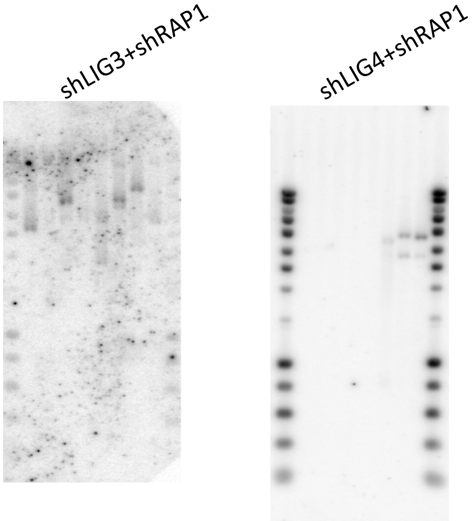

21 probe

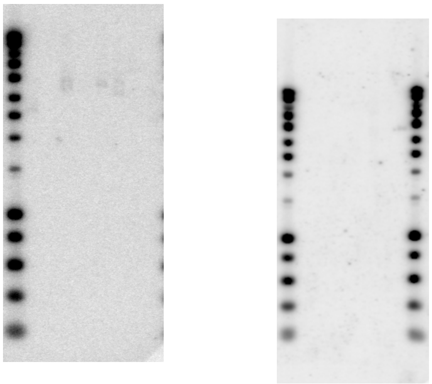

X probe

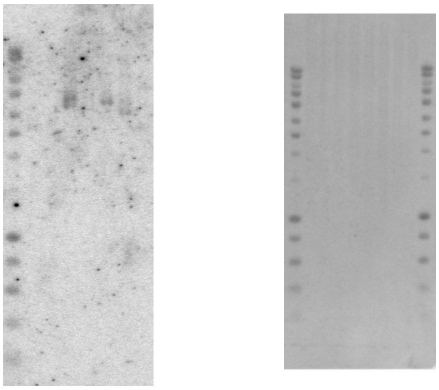

30 15

Figure 2

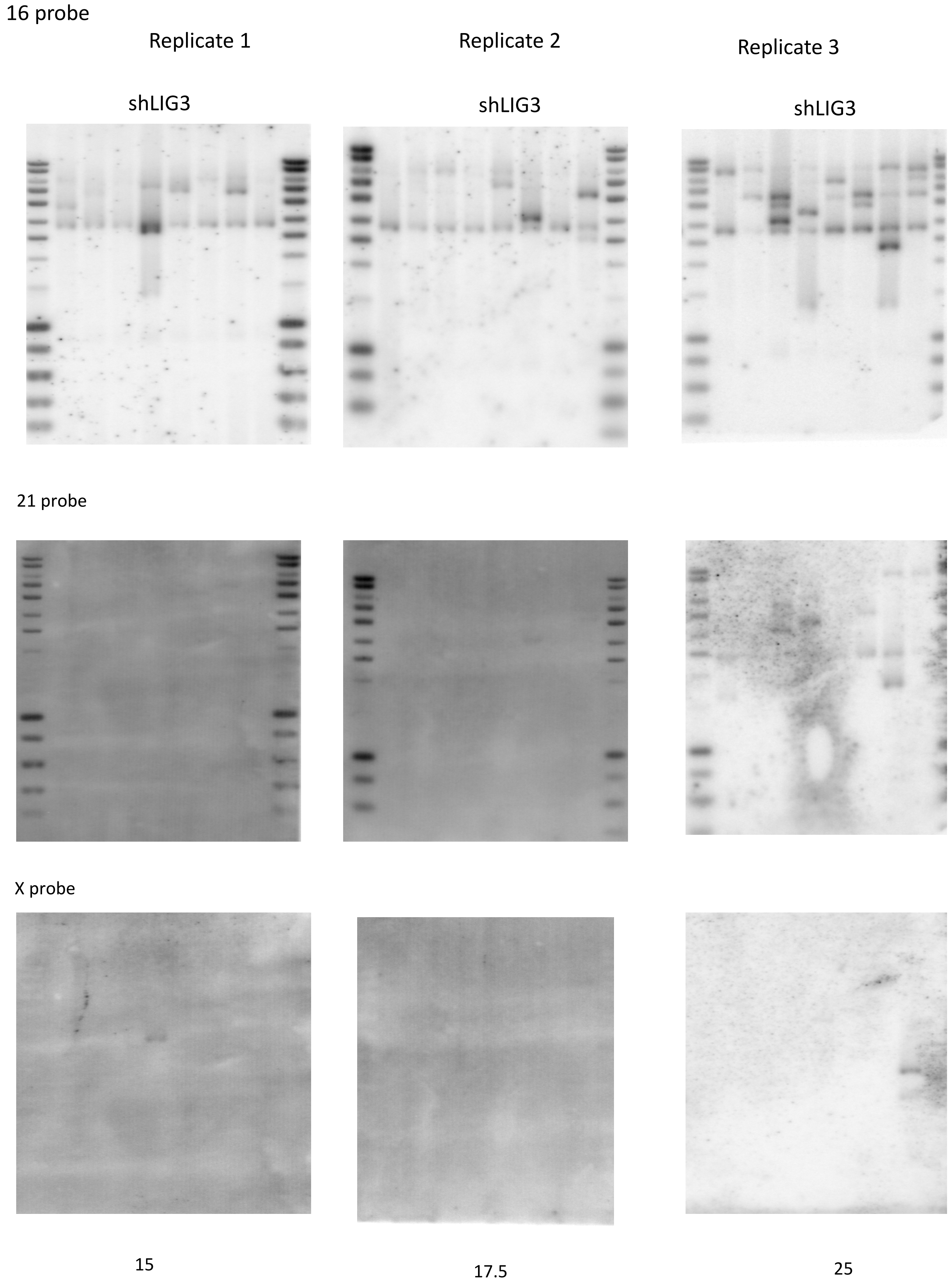
16 probe
Replicate 1
Replicate 2
Replicate 3
shLIG3
shLIG3
shLIG3
21 probe
X probe
15
17.5
25

Figure 2

Replicate 1 Replicate 2 Replicate 3

16 probe

shLIG4 shLIG4 shLIG4

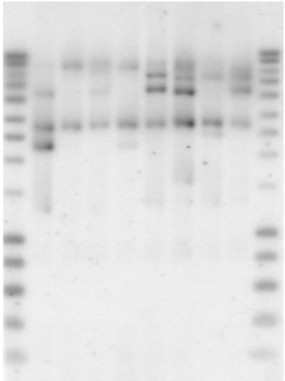

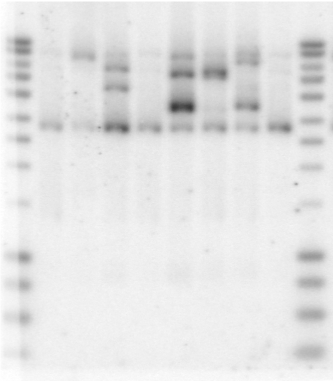

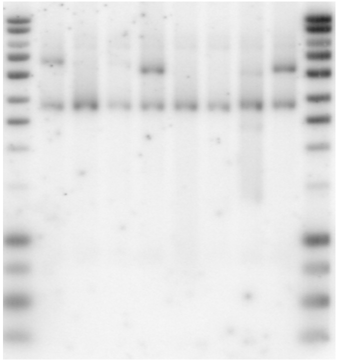

21 probe

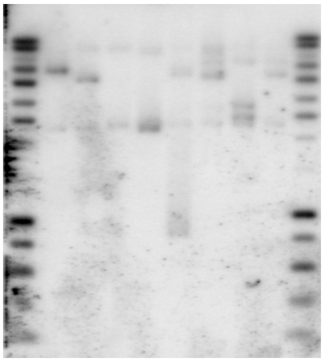

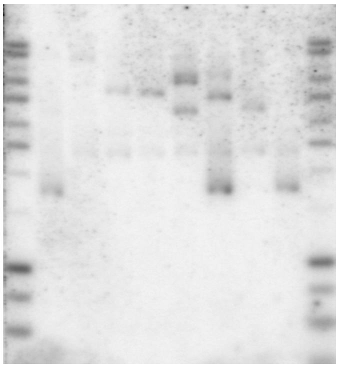

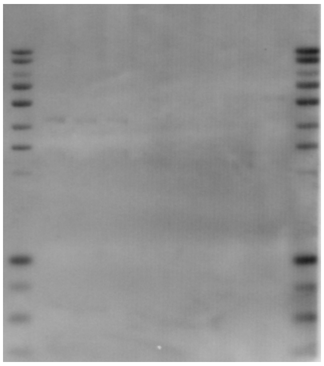

X probe

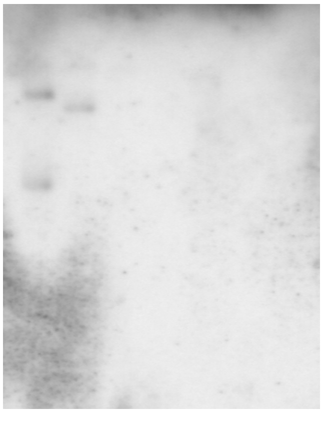

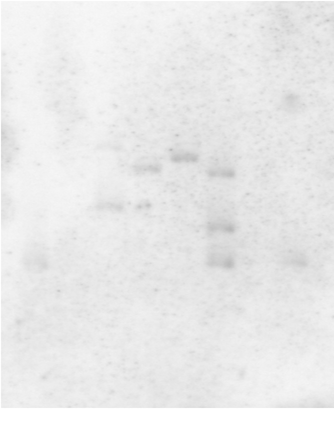

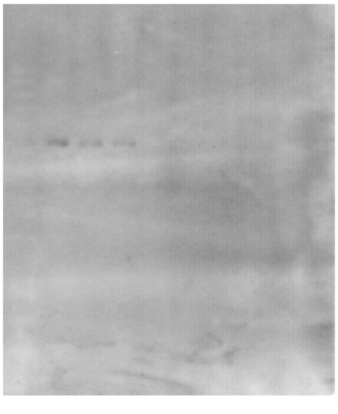

22.5 20 12.5

Figure 2

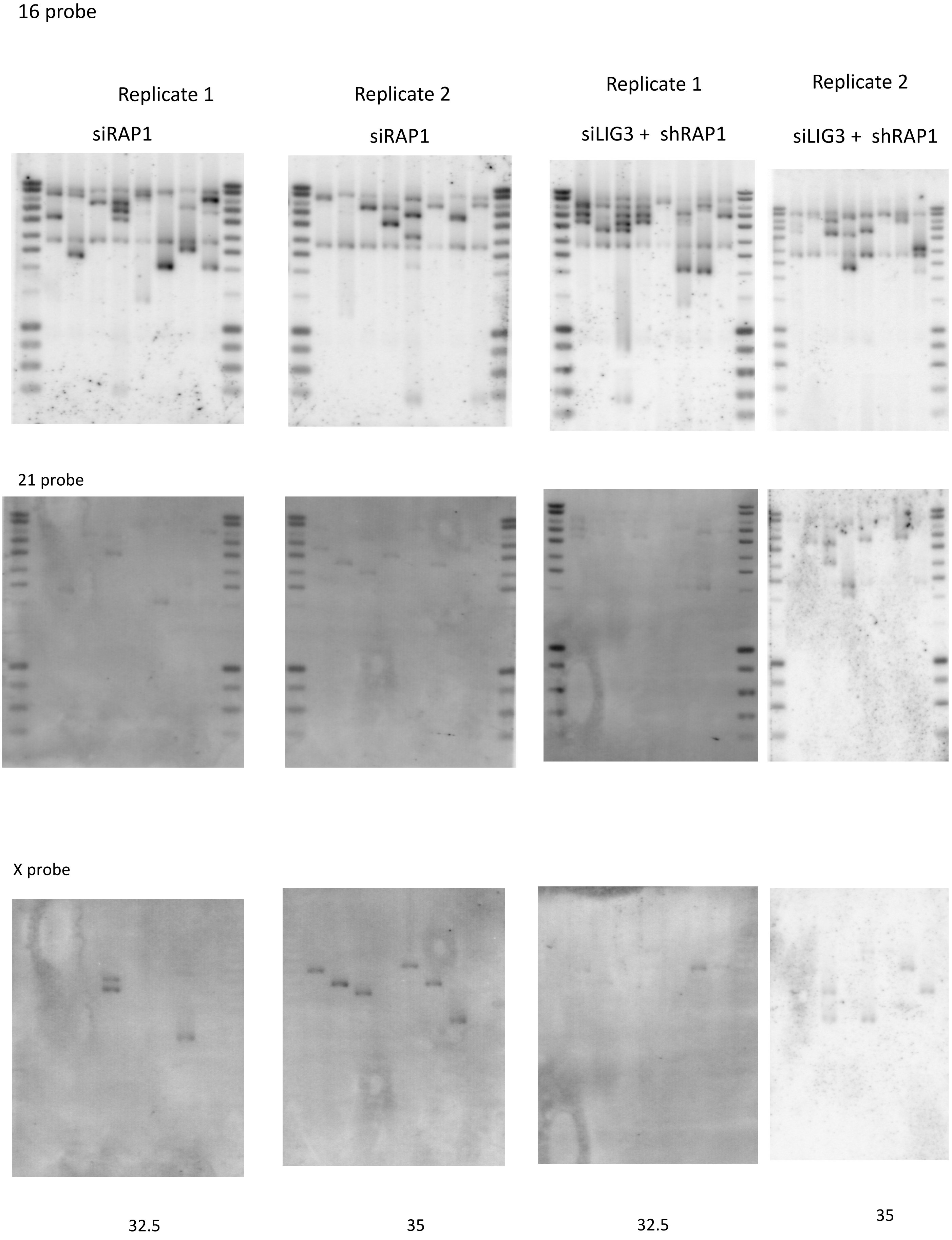
16 probe
Replicate 1
siRAP1
Replicate 2
siRAP1
Replicate 1
siLIG3 + shRAP1
Replicate 2
siLIG3 + shRAP1
21 probe
X probe
32.5
35
32.5
35

Figure 2

16 probe

Replicate 1

siLIG4 + shRAP1

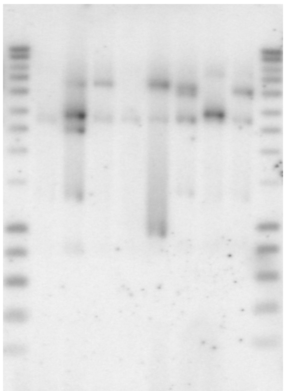

Replicate 2

siLIG4 + shRAP1

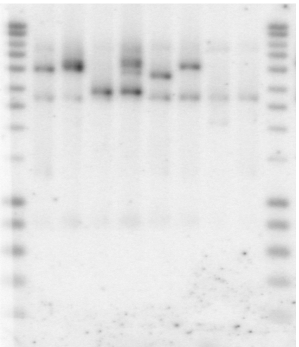

Replicate 3

siLIG4 + shRAP1

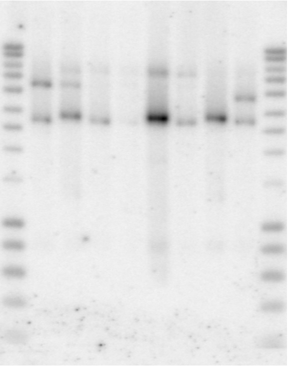

21 probe

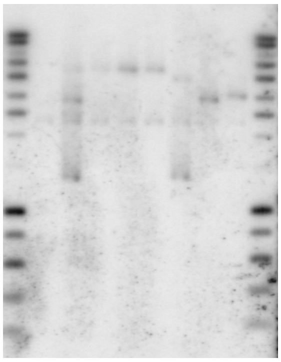

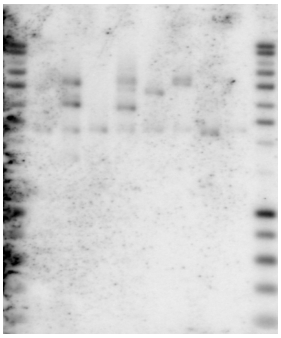

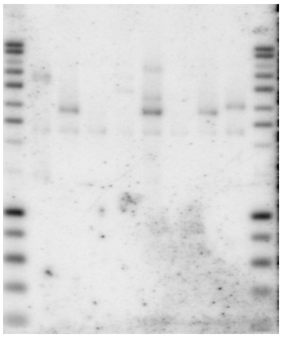

X probe

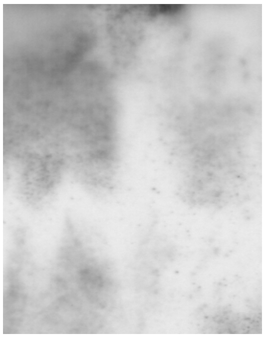

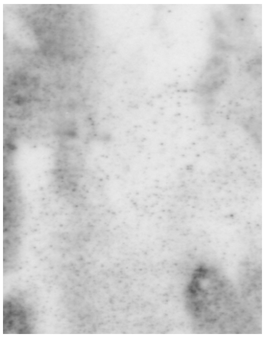

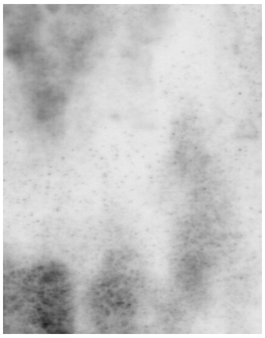

22.5

17.5

15

Figure 2

Replicate 1

16 probe

ΔRCT+shRAP1 shRAP1 + ΔMYB shRAP1 + RAP1

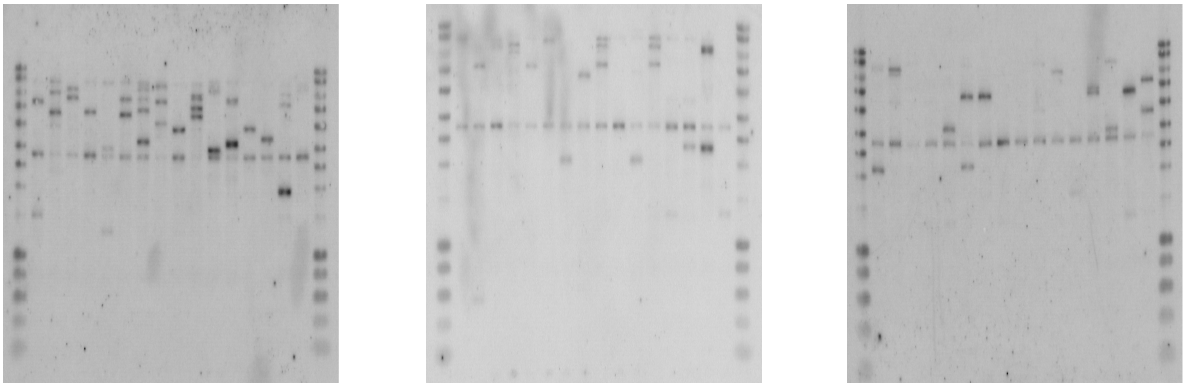

21 probe

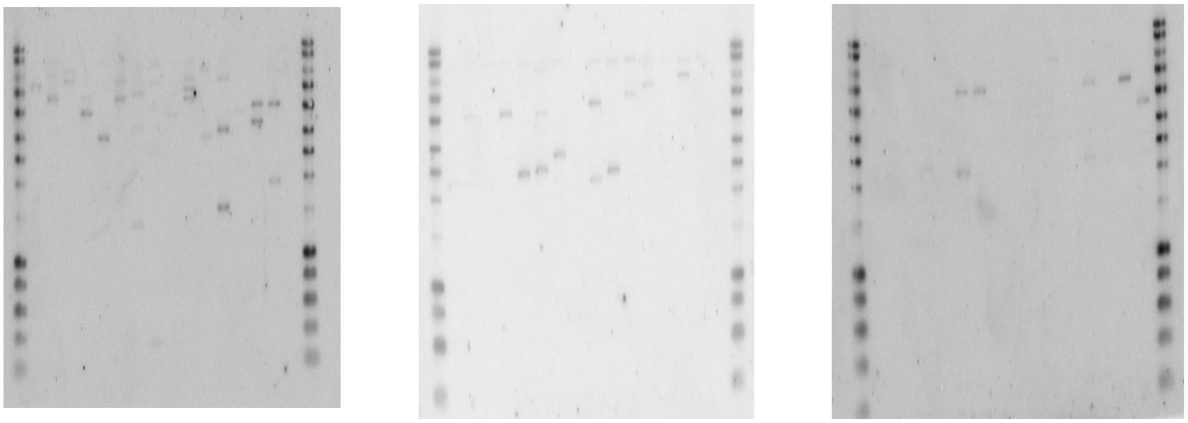

X probe

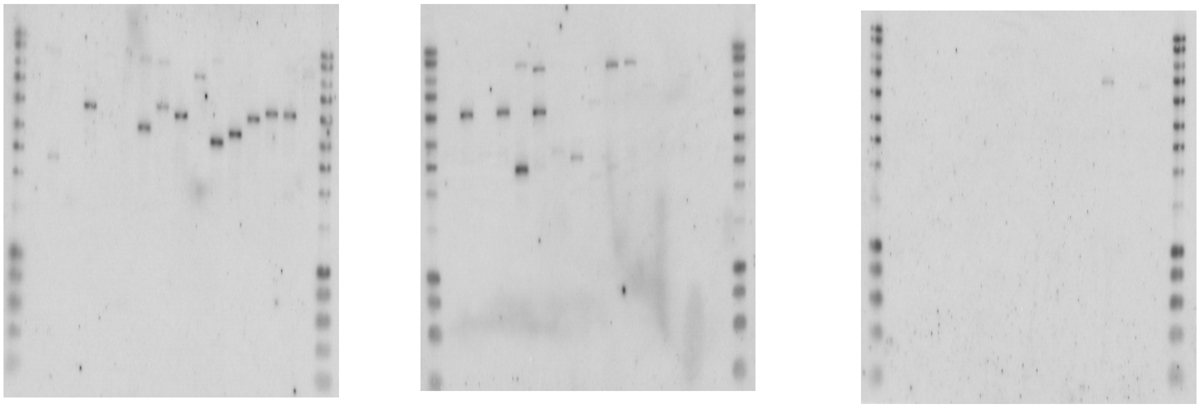

45 25 20

Figure 2

Replicate 2

16 probe

ΔRCT+shRAP1 shRAP1 + ΔMYB shRAP1 + RAP1

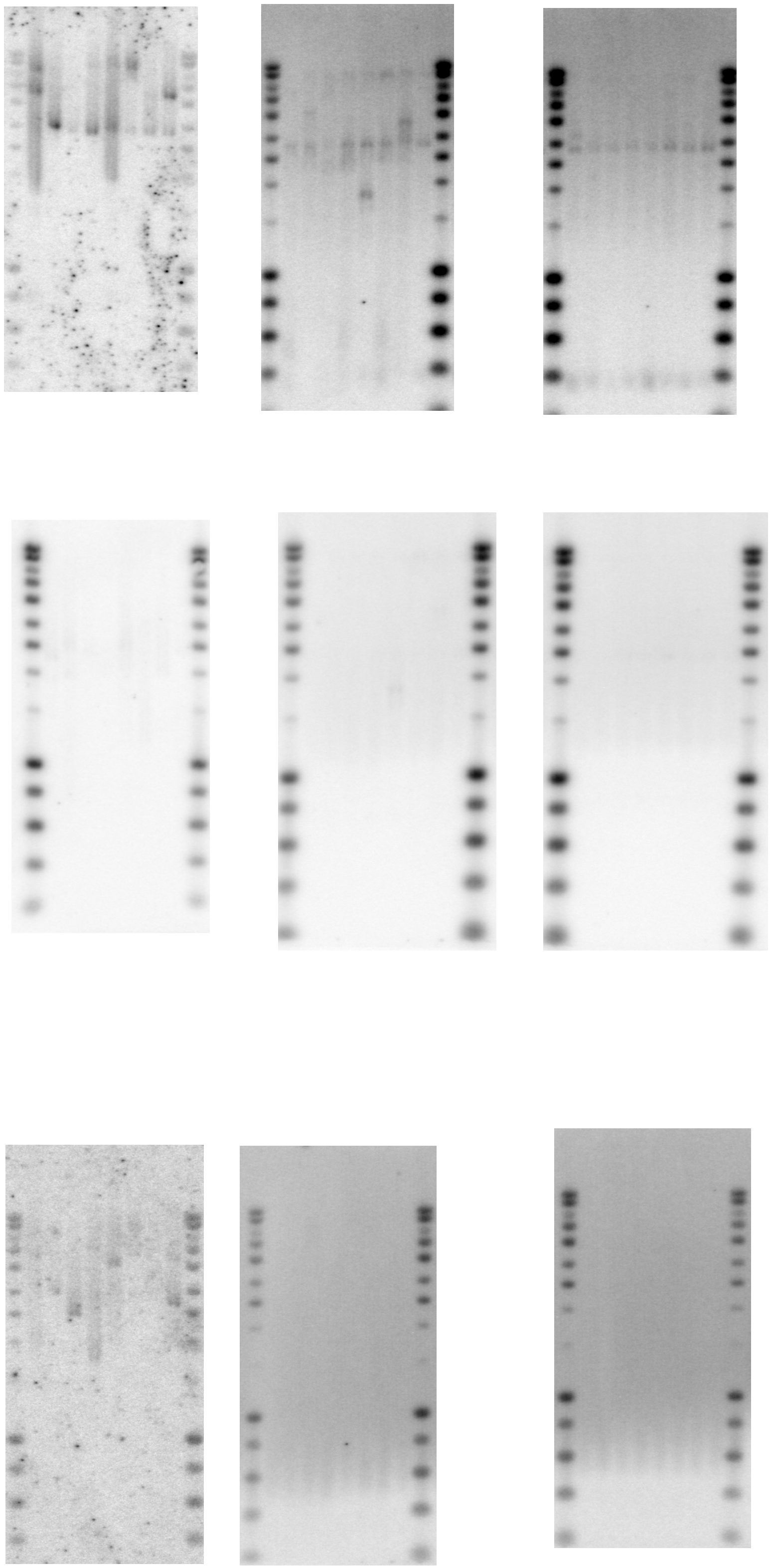

21 probe

X probe

30 12.5 10

Figure 2
e-f Replicate 3

16 probe

ΔRCT+shRAP1 | shRAP1 + ΔMYB | shRAP1 + RAP1

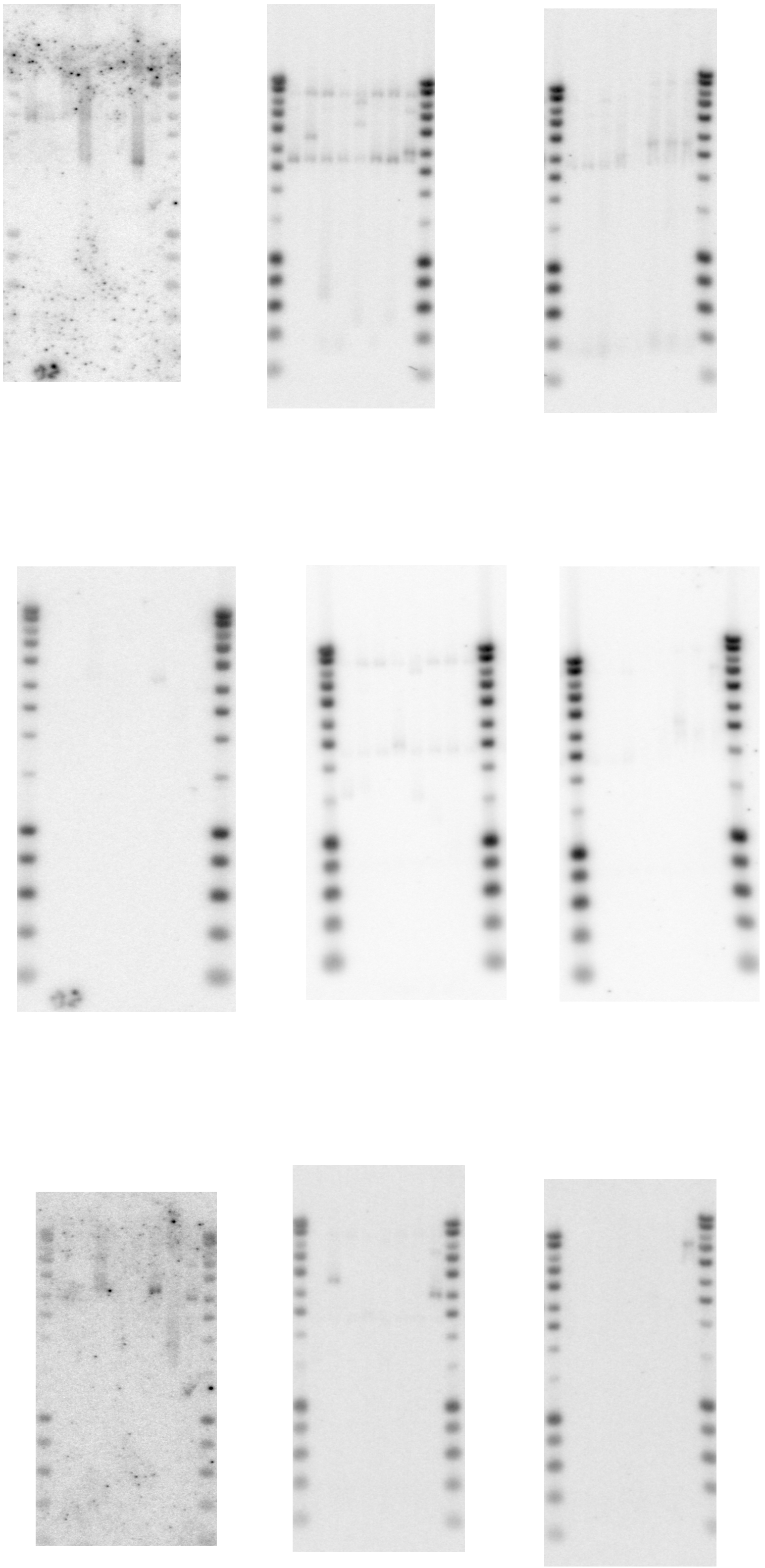

21 probe

X probe

27.5 | 27.5 | 20

Figure 4

a

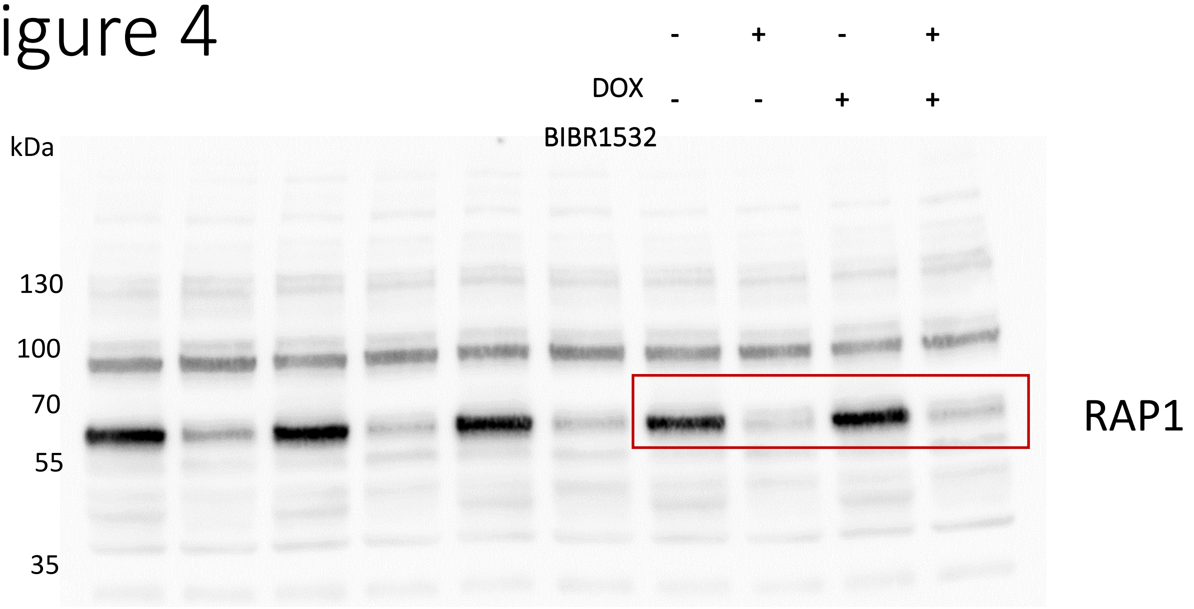

- + - +
DOX
- - + +
BIBR1532
kDa
130
100
70
55
35
RAP1

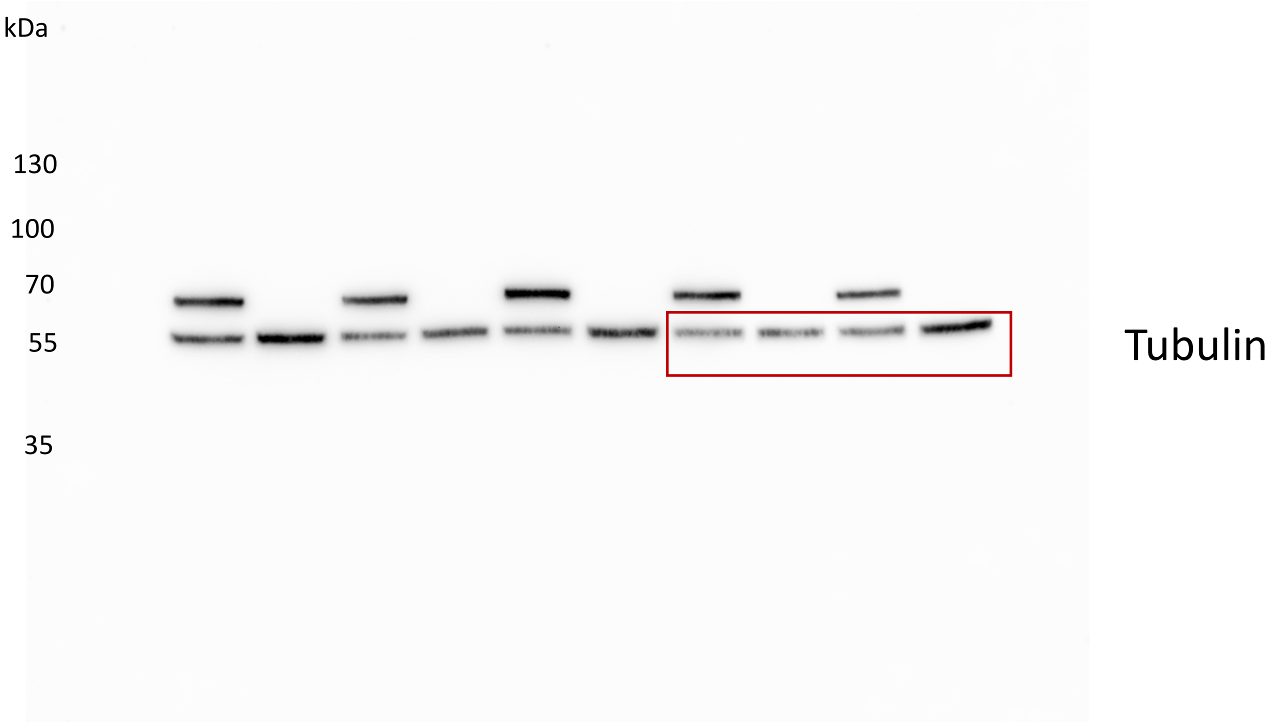

kDa
130
100
70
55
35
Tubulin

Figure 4

c

Replicate 1

Replicate 2

16 probe

DMSO -DOX

DMSO +DOX

BIBR -DOX

BIBR +DOX

BIBR -DOX

BIBR +DOX

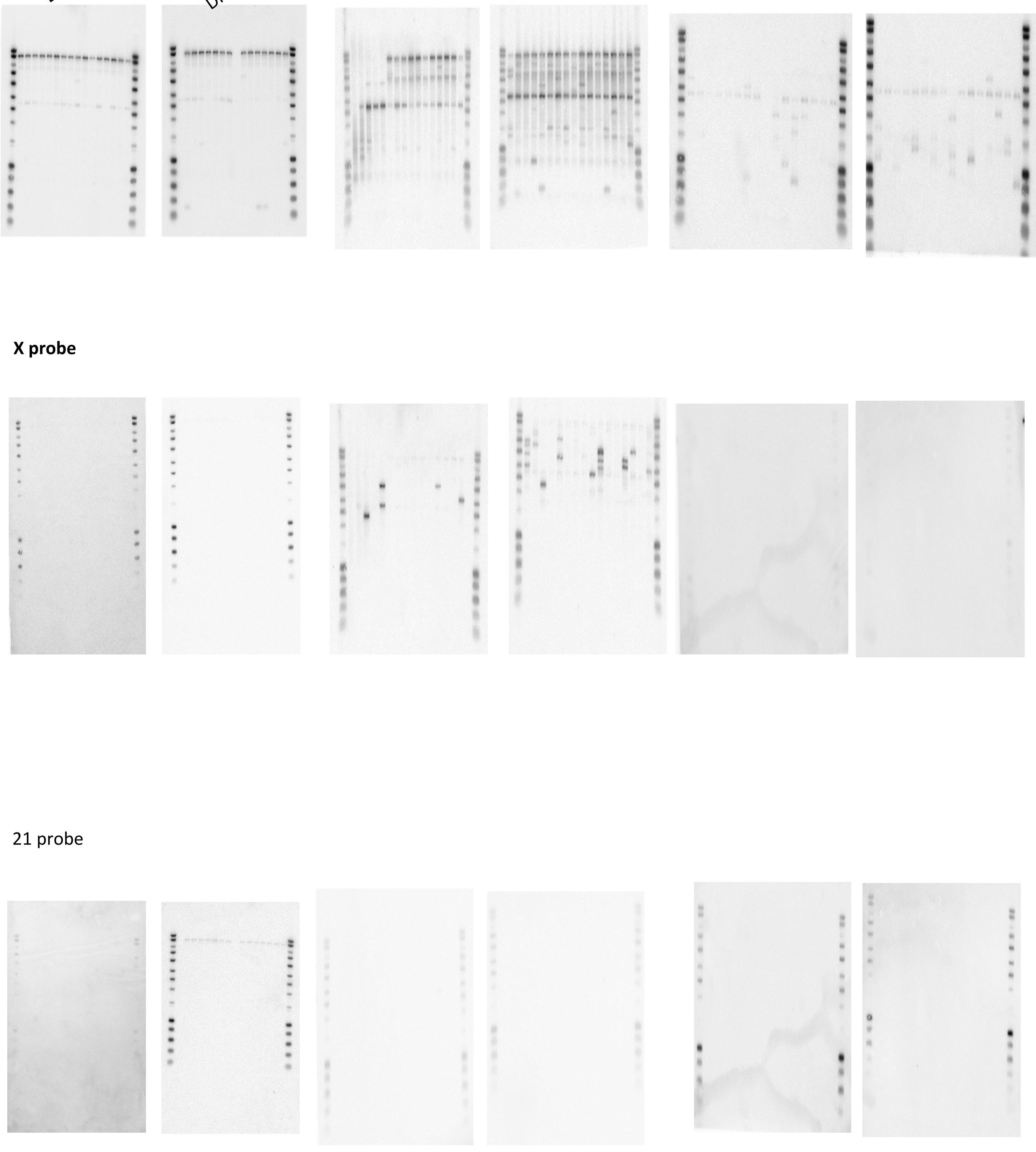

X probe

21 probe

6

8

21

48

17

32

Figure 4

c Replicate 1

16 probe

+BIBR +DOX+shControl | +BIBR -DOX+shLIG4 | +BIBR +DOX+shLIG3 | +BIBR +DOX+shLIG4

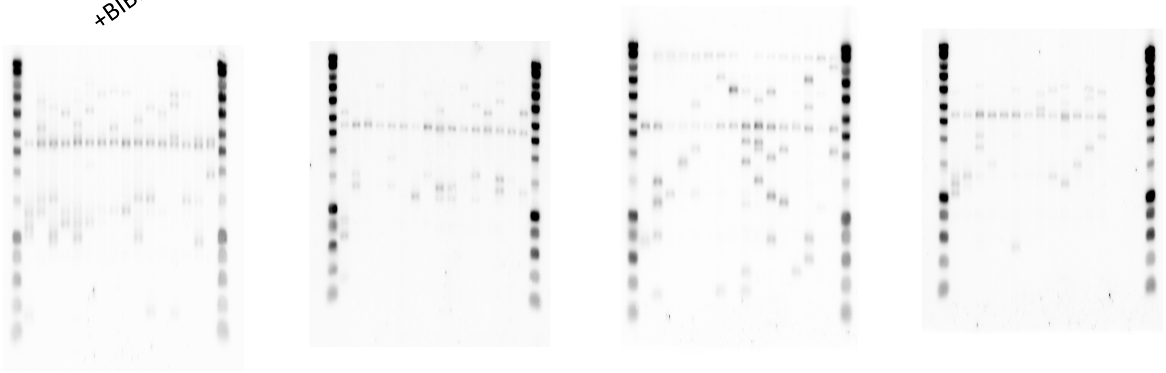

X probe

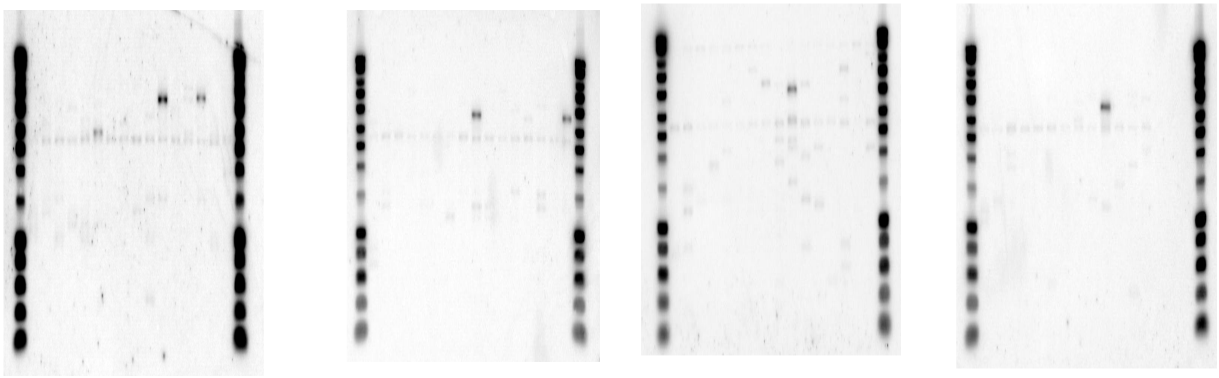

21 probe

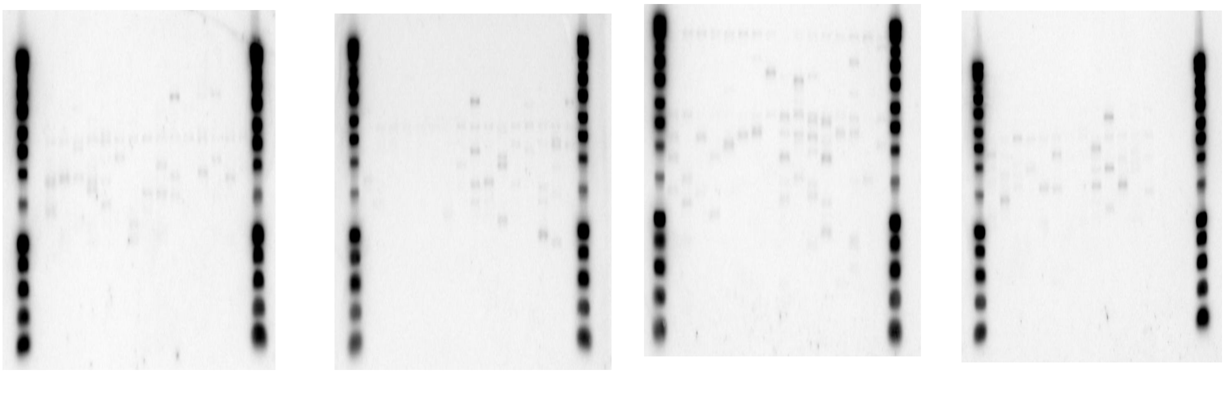

39 | 22 | 45 | 24

Figure 4

c Replicate 1

21 probe

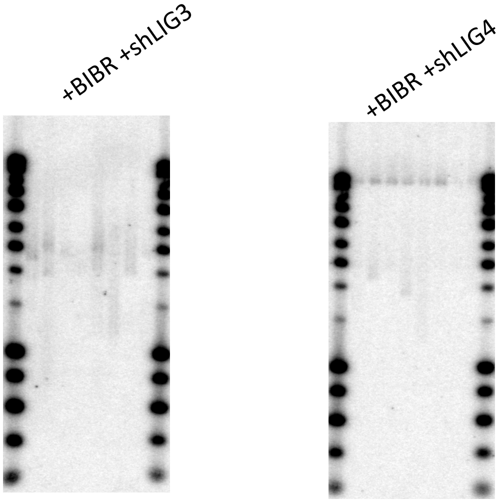

X probe

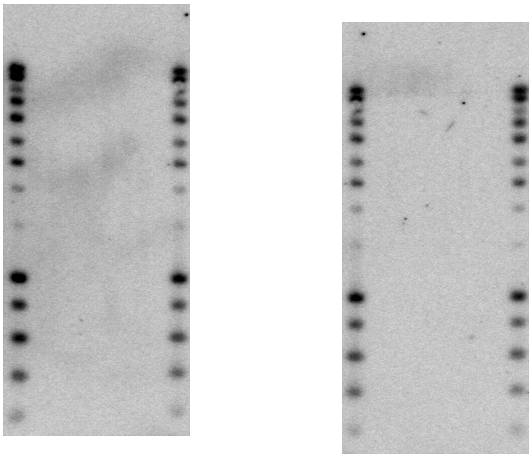

16 probe

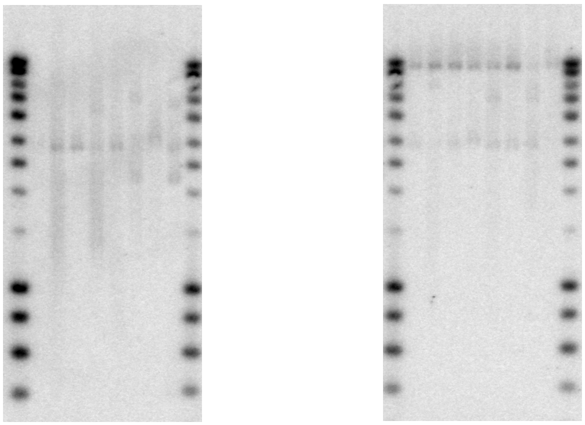

20

17.5

Figure 4

c Replicate 2

21 probe

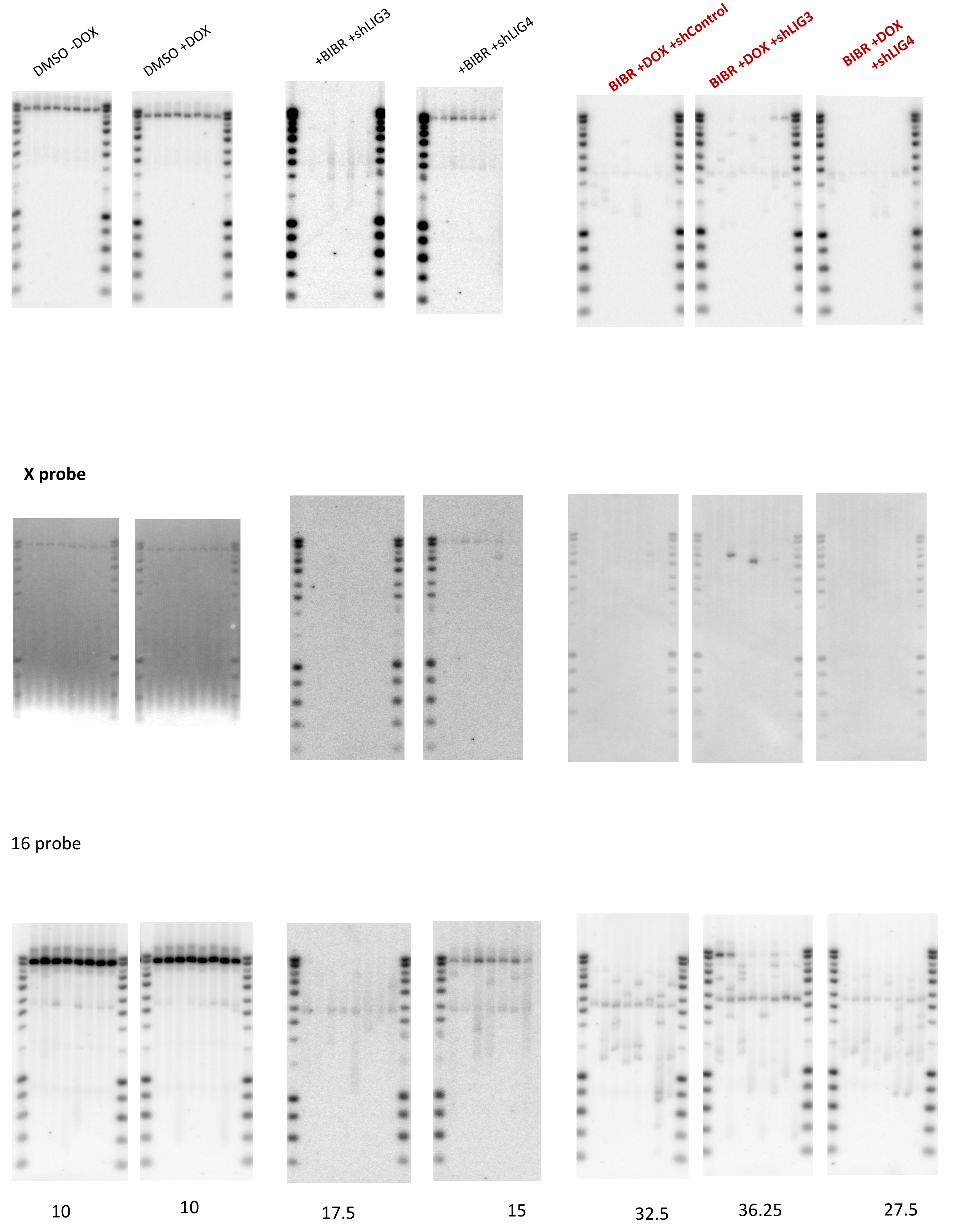

100 ng of DNA per PCR reaction

Figure 4

c Replicate 3

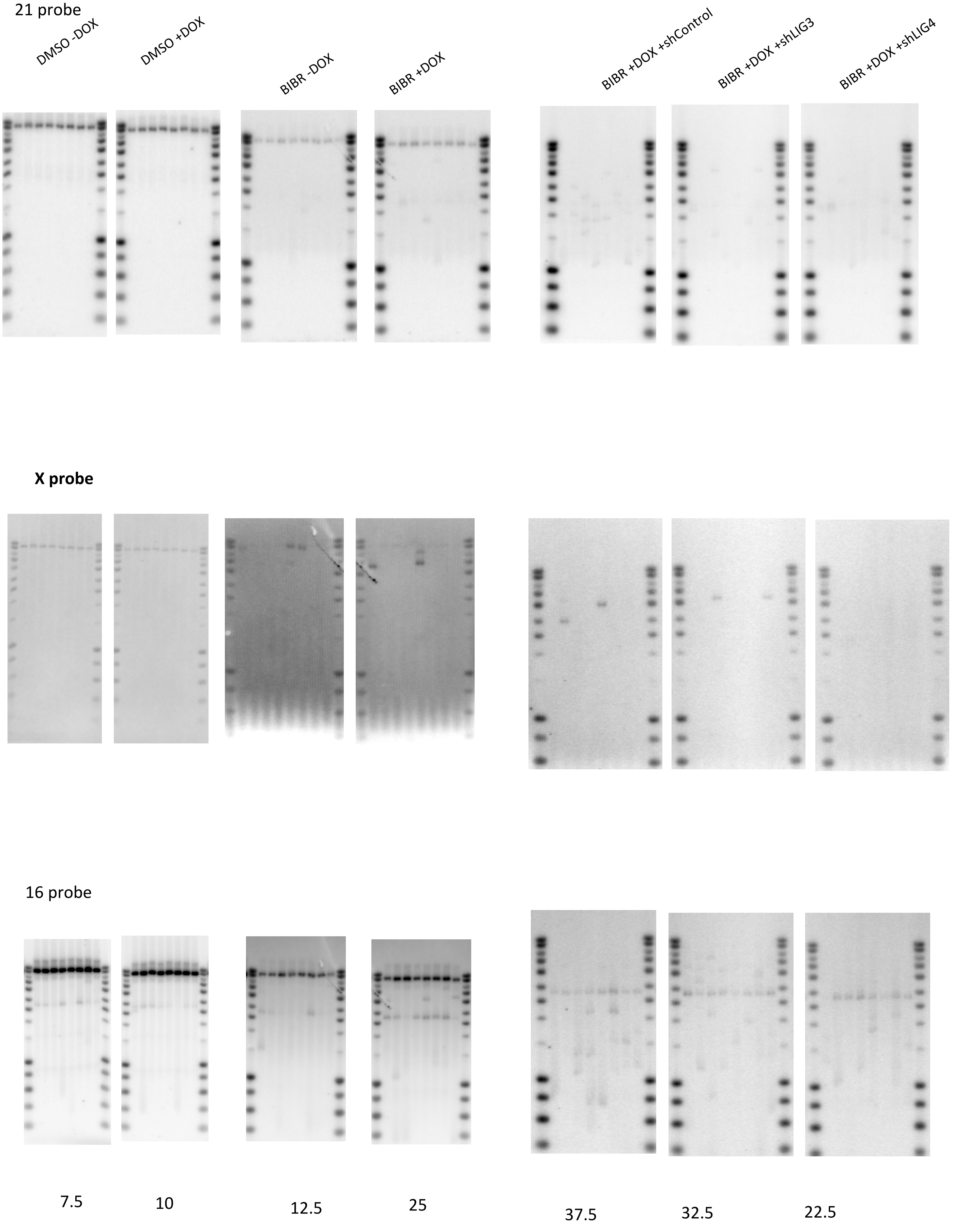

Figure 5
b

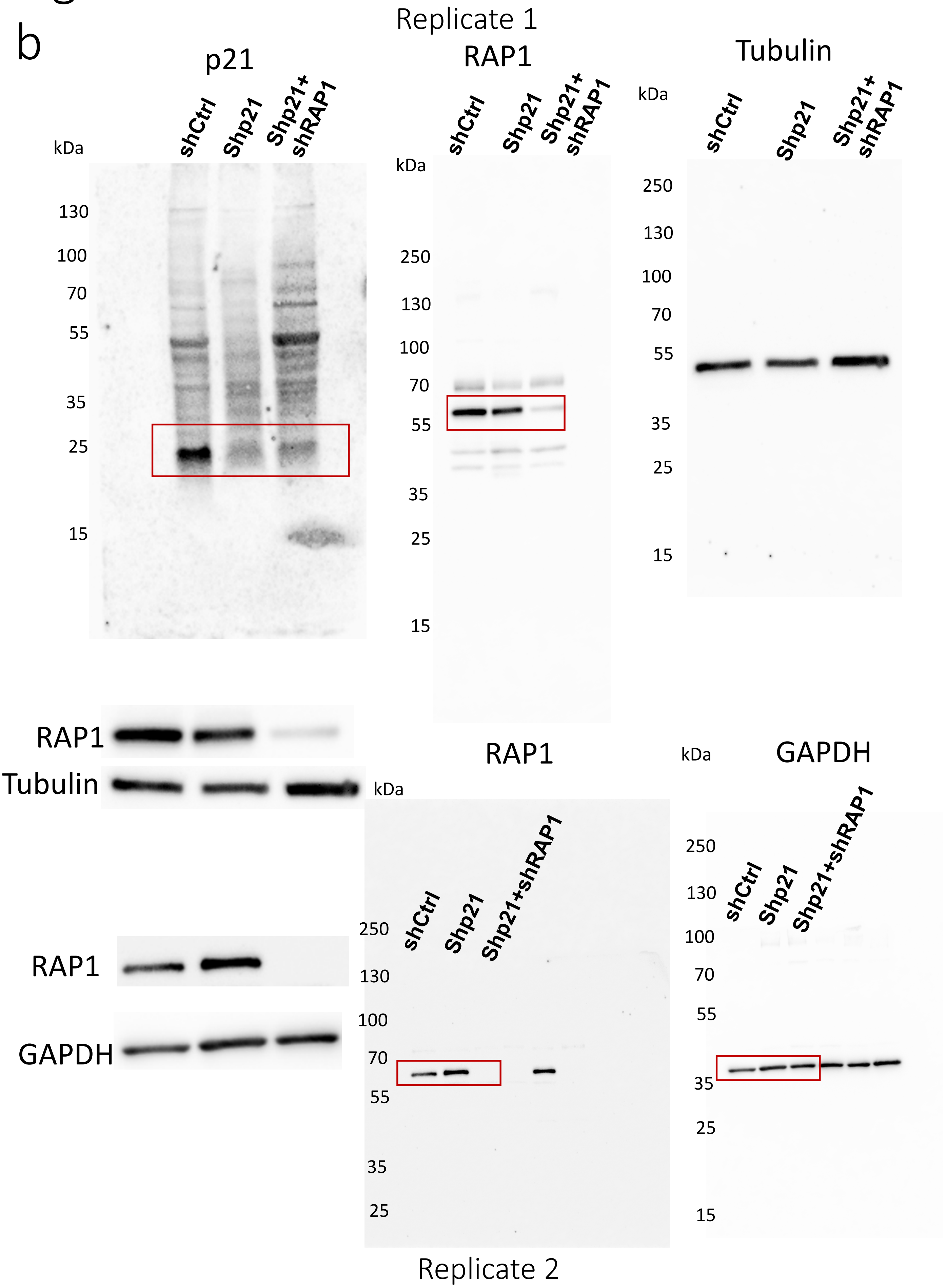
Replicate 1
p21
shCtrl
Shp21
Shp21+ shRAP1
kDa
130
100
70
55
35
25
15
RAP1
shCtrl
Shp21
Shp21+ shRAP1
kDa
250
130
100
70
55
35
25
15
Tubulin
kDa
shCtrl
Shp21
Shp21+ shRAP1
250
130
100
70
55
35
25
15
RAP1
Tubulin
RAP1
GAPDH
RAP1
kDa
shCtrl
Shp21
Shp21+shRAP1
250
130
100
70
55
35
25
kDa
GAPDH
shCtrl
Shp21
Shp21+shRAP1
250
130
100
70
55
35
25
15
Replicate 2

EVF1

a

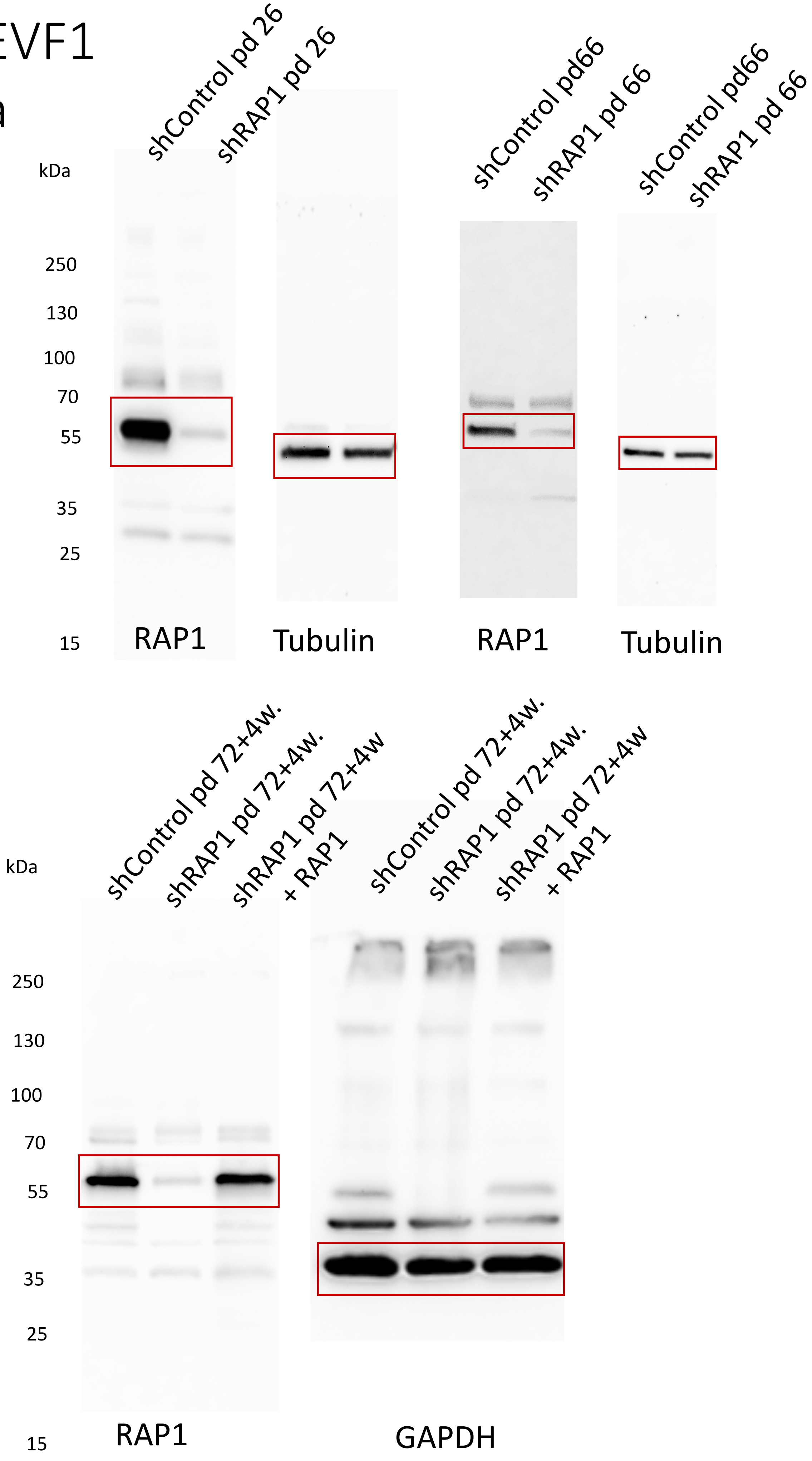

shControl pd 26
shRAP1 pd 26
shControl pd66
shRAP1 pd 66
shControl pd66
shRAP1 pd 66
kDa
250
130
100
70
55
35
25
15
RAP1
Tubulin
RAP1
Tubulin
shControl pd 72+4w.
shRAP1 pd 72+4w.
shRAP1 pd 72+4w
+ RAP1
shControl pd 72+4w.
shRAP1 pd 72+4w.
shRAP1 pd 72+4w
+ RAP1
kDa
250
130
100
70
55
35
25
15
RAP1
GAPDH

EVF2

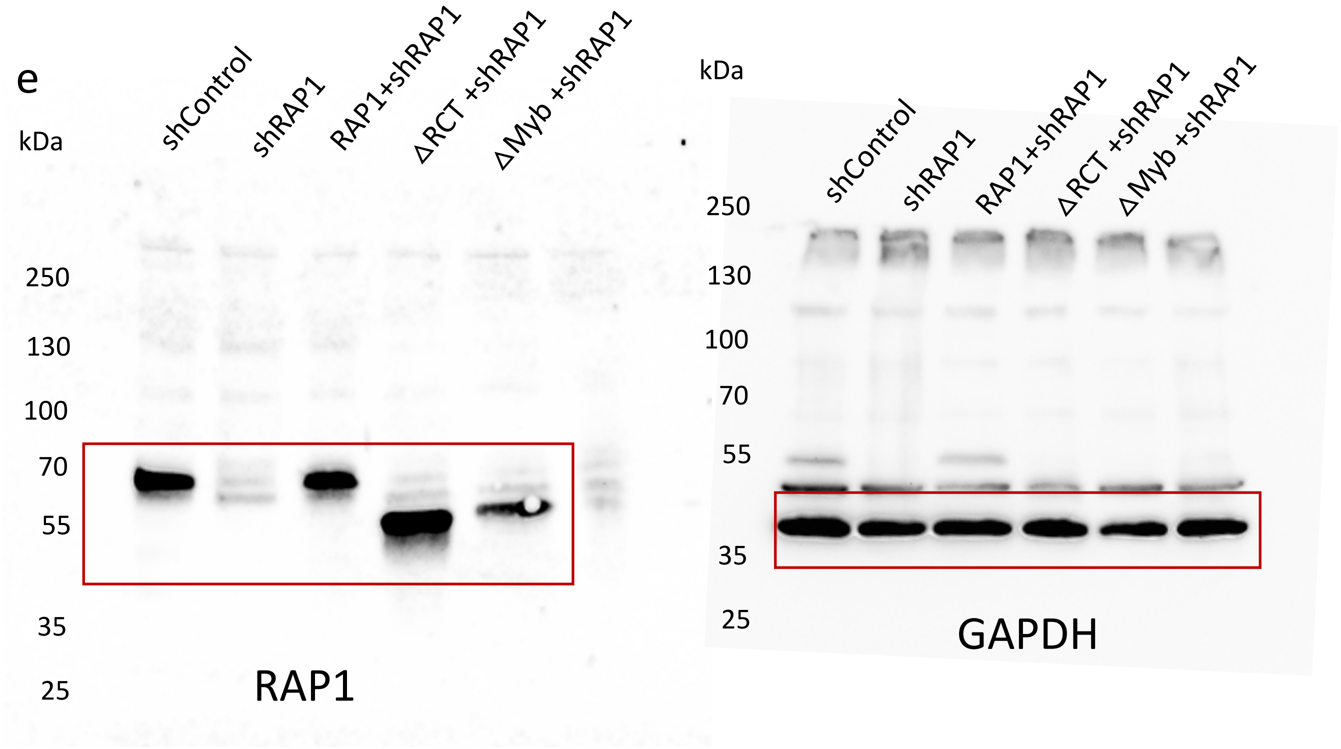

e
kDa
shControl
shRAP1
RAP1+shRAP1
ΔRCT +shRAP1
ΔMyb +shRAP1
250
130
100
70
55
35
25
RAP1
kDa
250
130
100
70
55
35
25
GAPDH

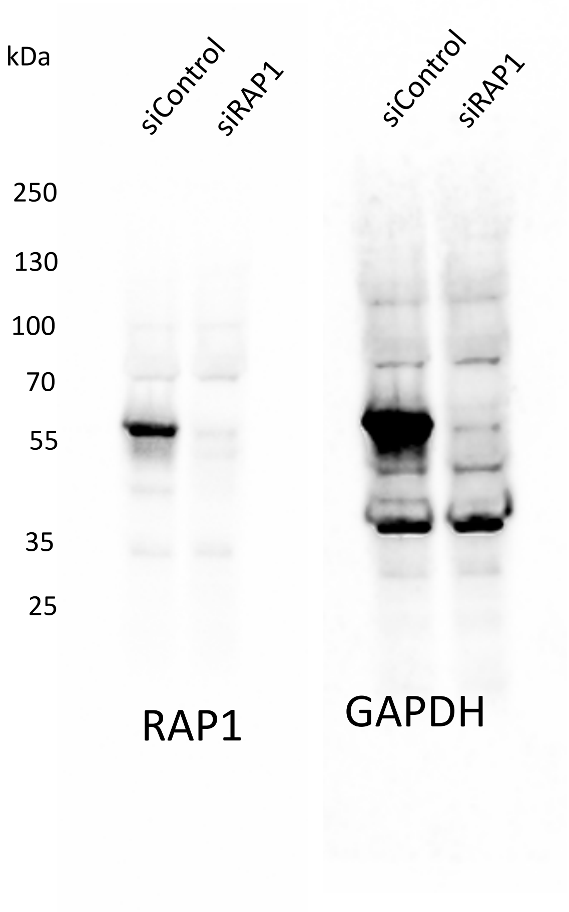

kDa
siControl
siRAP1
250
130
100
70
55
35
25
RAP1
GAPDH

EVF2

e

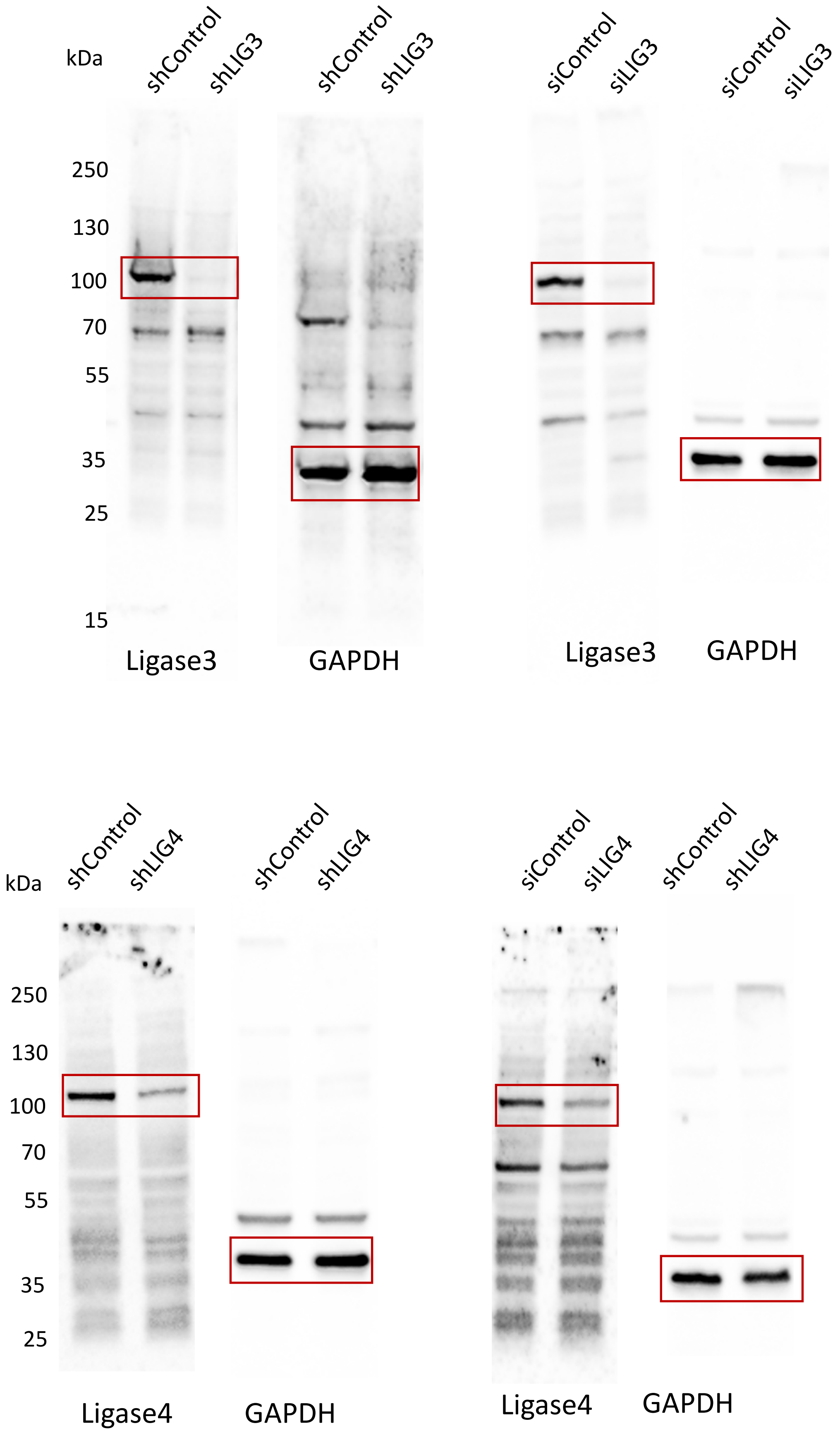

kDa
shControl
shLIG3
shControl
shLIG3
siControl
siLIG3
siControl
siLIG3
250
130
100
70
55
35
25
15
Ligase3
GAPDH
Ligase3
GAPDH
kDa
shControl
shLIG4
shControl
shLIG4
siControl
siLIG4
shControl
shLIG4
250
130
100
70
55
35
25
Ligase4
GAPDH
Ligase4
GAPDH

EVF3

c

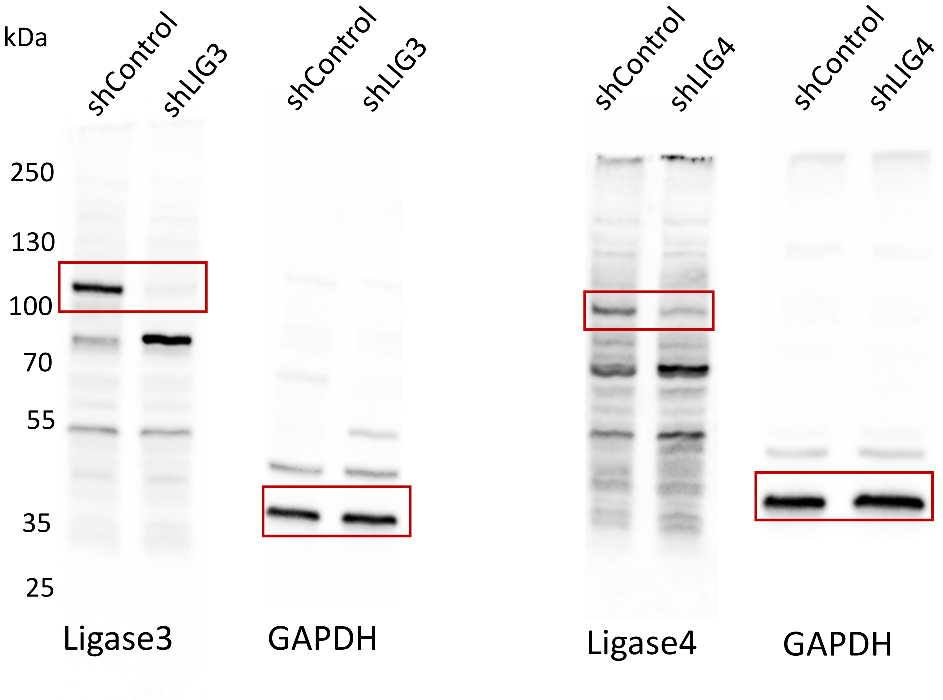
kDa
shControl
shLIG3
shControl
shLIG3
shControl
shLIG4
shControl
shLIG4
250
130
100
70
55
35
25
Ligase3
GAPDH
Ligase4
GAPDH

d

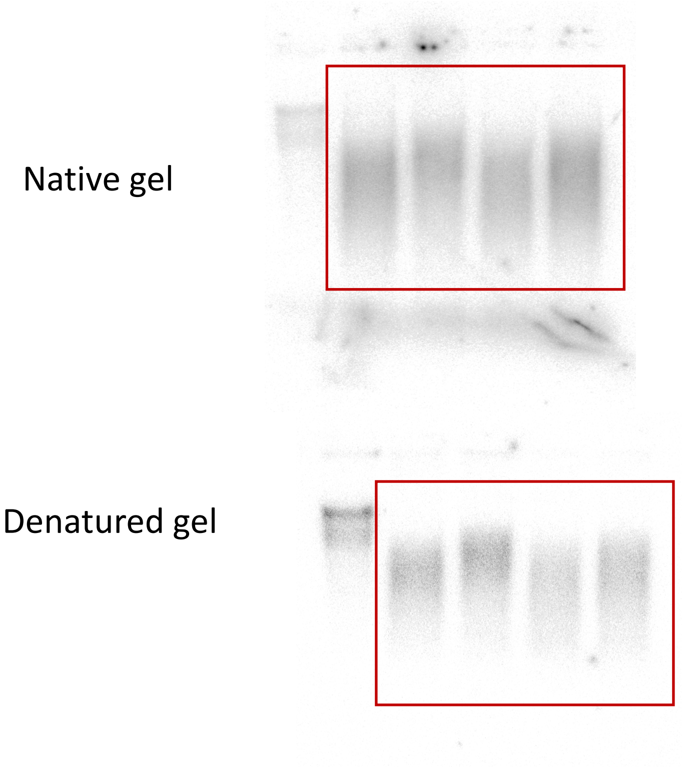
Native gel
Denatured gel
